# Predicting the Key Properties of a Modified Product to Pre-Select a Pluronic F127 Modification Scheme for Preparing High-Quality Nano-Micelles

**DOI:** 10.3390/polym17030349

**Published:** 2025-01-27

**Authors:** Jizheng Song, Yu Hu, Shiyu Yang, Dexue Liu, Yiider Tseng, Lingjun Li

**Affiliations:** 1 College of Pharmacy, Shandong University of Traditional Chinese Medicine, Jinan 250355, China; 60030119@sdutcm.edu.cn (J.S.);; 2 Innovative Institute of Chinese Medicine and Pharmacy, Shandong University of Traditional Chinese Medicine, Jinan 250355, China; ytseng@sdutcm.edu.cn

**Keywords:** pre-selecting suitable hydrophobic groups, hydrophile–lipophile balance, critical micelle concentration, Gibbs free energy, pharmaceutical evaluation methods

## Abstract

Hydrophobic modification alters the properties of Pluronic F127 to form micelles more efficiently and enhances its drug-loading capacity. However, selecting the appropriate hydrophobic group for modification is laborious. In this paper, we propose an efficient approach for predicting key parameters to select hydrophobic groups for F127 modification prior to synthesis, in order to improve the formability and stability of the micelles. The results of nuclear magnetic resonance and isothermal titration calorimetry were utilized to establish a function for predicting the hydrophile–lipophile balance, critical micelle concentration, and Gibbs free energy of the products based on the structure of raw material. These predicted values can assist us in selecting suitable hydrophobic groups for F127 modification. Subsequently, we successfully tested our method and validated our work using pharmaceutical evaluation methods, such as appearance observation, particle size measurement, drug loading determination, equilibrium binding rate assessment, storage stability testing, and the plotting of accumulation release curves. Therefore, we suggest that our work could provide a model linking the molecular structure to properties, with the purpose of pre-selecting modification products that have advantages in micelle preparation. This can facilitate the application of F127 in preparing nano-micelles.

## 1. Introduction

Various drug delivery vehicles have the potential to enhance the water solubility of the drugs and reduce their severe side effects. Among them, micelles have advantages, including easy preparation, the ability to encapsulate multiple drugs, and the capacity to shield drugs from degradation and destruction [1]. Amphiphilic polymers with excellent properties serve as the cornerstone for obtaining nano-micelles with superior characteristics. For this reason, scientists have been continuously developing high-quality polymers. In recent decades, Pluronic has been a key player in preparing micelles to deliver toxic or hydrophobic drug molecules to their sites of action through passive targeting [2,3,4]. However, some challenges remain, such as high critical micelle concentration (CMC) [5,6,7] and undesirable hydrophobic drug encapsulation efficiency (EE) [8,9]. To address these limitations, researchers have used hydrophobic materials or groups to modify Pluronic and enhance its effectiveness [10,11].

Currently, selecting hydrophobic groups to modify Pluronic F127 (F127) and preparing high-quality micelles is a laborious and imprecise task, relying heavily on trial-and-error methods [12]. A more streamlined evaluation guideline is needed to reflect the need for a more efficient and cost-effective method. The key challenge lies in how to link the structure of polymers with the quality of micelles through the properties of polymers, so that the modification and screening of polymers can be target-oriented. Therefore, identifying the parameters that can accurately represent the properties of modification products and devising a practical approach to obtain these parameters and establish prediction methods are the focal points of our work.

Hydrophile–lipophile balance (HLB), CMC, and changes in Gibbs free energy (ΔG) can quantify the properties of surfactants and amphiphilic polymers [13,14]. HLB can preliminarily assess the compatibility of drug–polymer [12,15], and polymers with low CMC readily form stable micelles [16]. In the meantime, according to the Second Law of Thermodynamics, when ΔG is negative, the formation of micelles is a spontaneous process [17]. The greater the absolute value of ΔG during this process, the easier it is for the polymer to aggregate into micelles [18].

Therefore, obtaining the prediction values of HLB, CMC, and ΔG could aid in the design and synthesis of the amphiphilic polymer. To date, research works have used the emulsifying process, cloud point, polarity index, partition coefficient, and nuclear magnetic resonance (NMR) to obtain HLB [19,20,21], and some works have used conductivity, surface tension, refractive index, fluorescence probe, and isothermal titration calorimetry (ITC) to obtain the CMC [22,23,24]. HLB and CMC have been used to establish prediction models using quantitative structure–property relationships models or group contribution methods [24]. However, HLB values may not calculated due to the absence of a group contribution value [25], and these models are often applied to the prediction of small molecular surfactants [26,27]. In addition, these models have the problem that the method for detecting HLB and CMC is somewhat complicated [28,29]. As mentioned above, obtaining HLB, CMC, and ΔG with convenient methods is more helpful in evaluating the polymers. Among these methods, NMR and ITC are efficient ways to acquire the HLB, CMC, and ΔG of surfactants and amphiphilic polymers, respectively.

The research on the methods to predict the HLB, CMC, and ΔG of surfactants mainly consists of two sections: First, establishing the prediction method. Second, the agreement between the predicted and experimentally obtained values must be verified. As mentioned above, the formability of micelles is correlated with the structure of surfactants and amphiphilic polymers through their properties. Therefore, in the next step, we need to consider how to link the structure of polymers, the properties of polymers, and the outcomes of formulations together, in order to establish a pre-selecting method for polymer synthesis schemes specifically tailored for the preparation of micelles.

Based on these grounds and our previous studies [30,31,32], we propose an efficient approach for pre-selecting a hydrophobic group for F127 modification to prepare high-quality nano-micelles in this work. At first, we obtained the hydrogen spectra and the calorimetric curves by NMR spectroscopy and ITC, respectively. Then, these results were used to establish prediction models by quantitative structure–activity relationships, which were subsequently employed to predict the HLB, CMC, and ΔG of the modification products. These three parameters could help us select suitable hydrophobic groups for F127 modification. Thereafter, we verified the correlation between polymer properties and the formability and stability of micelles using pharmaceutical evaluation indicators. Numerous studies have demonstrated that curcumin (CUR) possesses a wide range of pharmacological effects, such as anti-inflammatory, antioxidant, anti-aging, antibacterial, and anticancer. It is a drug component with extensive efficacy and high safety [33,34,35]. Therefore, we chose curcumin as the model drug to evaluate the pharmaceutics of the pre-selected product. The appearance, particle size, drug loading (DL), equilibrium binding rate, storage stability, and release profile were used as indexes to evaluate the formability of modified F127 and the stability of its micelles. In this way, it might serve as a guideline for a pre-selecting synthesis scheme for amphiphilic polymers to prepare high-quality nano-micelles.

## 2. Materials and Equipment

CUR was obtained from J&K Scientific (Beijing, China) with a purity of 98%. Cyano-modified silica was obtained from Macherey-Nagel (Düren, Germany). The polymers, poly-caprolactone (PCL, molecular weights (MW): 2000 Dalton (Da), 3500 Da and 5300 Da), poly-lactic acid (PLA, MW: 2000 Da, 3500 Da and 5000 Da), and poly-lactic-co-glycolic acid (PLGA, MW: 2500 Da and 4700 Da), were sourced from Daigang Biomaterial Co., Ltd. (Jinan, China). The purities of following chemical reagents are all analytical grade: F127, polyethene glycol 400 (PEG-400), 1-(3-dimethylaminopropyl)-3-ethylcarbodiimide (EDC), 4-dimethylaminopyridine (DMAP), triethylamine (TEA), dimethyl formamide (DMF), sodium dodecyl sulfate (SDS), dimethyl sulfoxide (DMSO), and α-cyano-4-hydroxycinnamic acid (CAHC) were all obtained from Aladdin Industrial Corporation (Shanghai, China). Span-60, Span-20, EL-35, Tween-80, Tween-20, and methanol were purchased from Sinopharm Chemical Reagent Co., Ltd. (Shanghai, China). We used various equipment for analysis: ITC MicroCal 200 was manufactured by General Electric Company (Boston, MA, USA). The rotary evaporator was purchased from IKA Reagent Co., Ltd. (Staufen, Germany). NMR was produced by Bruker (Rheinstetten, Germany). High-performance liquid chromatography (HPLC) 1260 was made by Agilent Reagent Co., Ltd. (Palo Alto, CA, USA). Matrix-assisted laser desorption/ionization time-of-flight mass spectrometry (MALDI-TOF-MS) was made by Bruker Daltonics Inc. (New York, NY, USA). The dynamic light scattering laser particle size analyzer (DLS) was produced by Malvern Instruments (Malvern, UK). Transmission electron microscopy JEM 1400 Plus (TEM) was made by JEOL Ltd. (Tokyo, Japan).

## 3. Methodology

### 3.1. Establishing a Novel Method to Pre-Select Hydrophobic Groups for F127 Modification

HLB, CMC, and ΔG are critical parameters for quantifying and characterizing the hydrophilicity of amphiphilic polymers and the difficulty of forming micelles. The innovation of our research lies in establishing predictive methods for these parameters to pre-select polymer modification schemes, with the aim of obtaining polymer materials that are more suitable for micelle preparation.

#### 3.1.1. Synthesis and Structural Confirmation of Model Polymers

**Figure 1:**
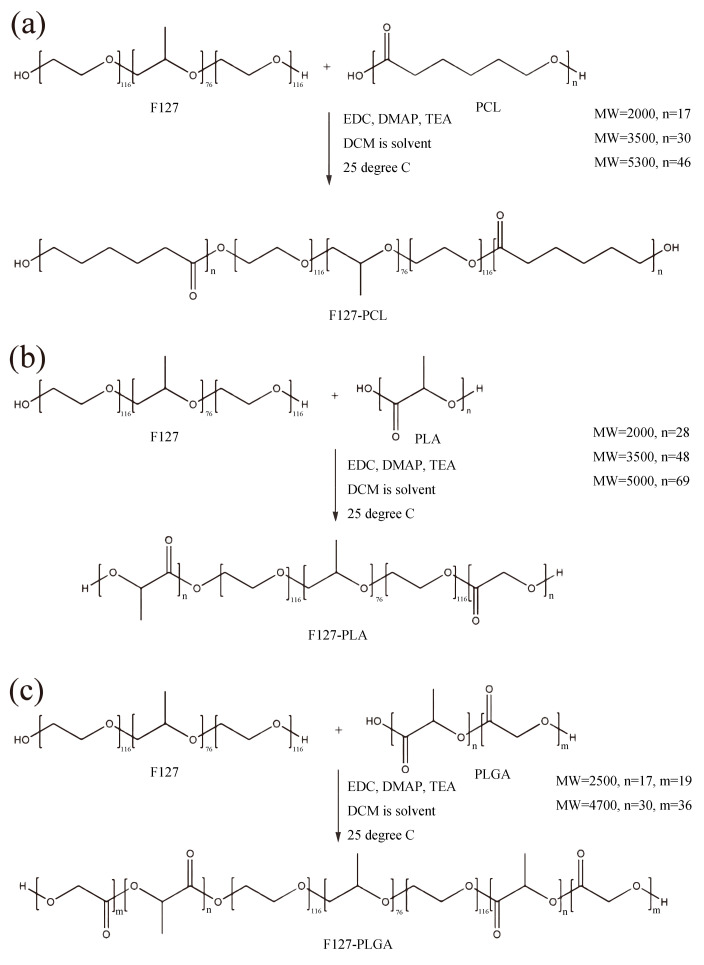
The synthetic route of F127-PCL (**a**), F127-PLA (**b**), and F127-PLGA (**c**).

Before synthesizing, F127 should be purified using Cyano-modified silica to remove small molecular impurities [36]. Supporting Information S1 presents the details of the purification process. The purification effect of F127 was confirmed using size exclusion chromatography (SEC) and MALDI-TOF-MS. The results are shown in Figures S1 and S2, and Table S1 in Supporting Information S1. All the polymer materials in this work were synthesized through an esterification reaction [30]. The synthetic routes are shown in Figure 1. The details are shown in Supporting Information S1.

NMR was used to identify the structures at 600 MHz, using deuterated chloroform as the solvent, to obtain the NMR hydrogen spectrometry (^1^H-NMR). Furthermore, the molecular weight distribution of F127 and its modification products were analyzed by MALDI-TOF-MS, providing a clear picture of the dispersity of these amphiphilic polymers. All the samples were analyzed on an autoflex speed MALDI-TOF mass spectrometer equipped with a 337 nm nitrogen laser. Spectra were obtained in the positive ion mode with an acceleration voltage of 20 kV. CAHA (50 mg/mL in DMF) was used as a matrix, and polymer samples were dissolved in DMF (10 mg/mL). They were mixed with a ratio of matrix: sample = 3:1, and 0.6 μL mixed solution was spotted on a MALDI sample plate.

#### 3.1.2. Using ^1^H-NMR and Compound Formula of Surfactant to Predict the HLB of Modified F127

In this section, we developed a novel method for predicting the HLB of synthetic products to guide amphiphilic polymer modification. First, ^1^H-NMR was used to determine the HLB of raw materials. Second, a formula was used to predict the HLB of modified F127. Finally, ^1^H-NMR was used again to obtain the HLB of modified F127, and the results were compared to the predicted values to verify the accuracy of the prediction method.

##### Establishing a Curve to Calculate the HLB of the Raw Materials

The ^1^H-NMR spectra of surfactants can be used to calculate their HLB [20,37]. Initially, 10 mg of Span-60, Span-20, EL-35, Tween-20, Tween-80, and PEG-400 were carefully weighed, and 700 μL of deuterium chloroform was added to dissolve the samples using ultrasound. Afterward, the solutions were transferred into the NMR sample tubes for the detection of their hydrogen spectra at 600 MHz. Since the chemical shifts of most hydrophobic groups are lower than 2.5 ppm and the chemical shifts of most hydrophilic groups are higher than 2.5 ppm, 2.5 ppm was adopted as a boundary value of the chemical shift to separate hydrophobic and hydrophilic groups. The MestreNova 6.1 software was used to process the spectra to obtain the peak areas for each chemical shift. Then, the total areas belonging to hydrophobic groups (∑A_o_) and hydrophilic groups (∑A_w_) were calculated. The relative coefficients of hydrophobic groups (H_o_) and hydrophilic groups (H_w_) were obtained by dividing ∑A_o_ and ∑A_w_ by the ∑A_w_, respectively. The hydrophilic coefficient (R) presents the ratio of hydrophilic groups in the amphiphilic polymer and can be calculated using Equation (1). Thus, the NMR spectra and HLB can be associated through R. A linear regression was performed with R as the x-coordinate and HLB as the y-coordinate.

(1) $$R=H_{w}/(H_{w}+H_{o})$$

##### Predicting the HLB of Modified Products Based on the HLB of Raw Materials and the Compound Formula

A total of 10 mg of F127, PCL (2000 Da, 3500 Da and 5300 Da), PLA (2000 Da, 3500 Da and 5000 Da), and PLGA (2500 Da and 4700 Da) were carefully weighed and dissolved in 700 μL of deuterium chloroform. After testing, the HLB was calculated as described above. We ignored changes in MW since these raw materials exhibited minimal variation in MW after modification. In this section, the mass of different surfactants in the compound formula [38] was replaced by MW. The modified formula is shown in Equation (2), which was used to predict the HLB of modified F127.

(2) $${\mathrm{HLB}}_{p}=\frac{M_{F127}}{M_{F127}+M_{\mathrm{hhc}}}\cdot {\mathrm{HLBF}}_{F127}+\frac{M_{\mathrm{hhc}}}{M_{F127}+M_{\mathrm{hhc}}}\cdot {\mathrm{HLB}}_{\mathrm{hhc}}$$

where the M_hhc_ denotes the MW of the hydrophobic group, M_F127_ denotes the MW of Pluronic F127, HLB_hhc_ denotes the HLB of the hydrophobic group, and HLB_F127_ denotes the HLB of Pluronic F127.

##### Comparing the Predicted and Measured Values to Evaluate the Accuracy of the HLB Predicting Method

Approximately 10 mg of modified F127 was carefully weighed, and 700 μL of deuterium chloroform was added to dissolve it using ultrasound. The solution was transferred into the NMR sample tube to obtain the hydrogen spectra at 600 MHz. The R value was calculated using the method described above. Then, R was substituted into the linear equation to obtain the tested HLB value for each material. Finally, the tested value was taken as the x-coordinate, and the predicted value was taken as the y-coordinate to fit the regression curve. The accuracy of the HLB predicting model was evaluated using the regression coefficient.

#### 3.1.3. Predicting the CMC of Modified F127

The CMC, a critical factor in the micellization process, correlates with molecular structure [39]. The molecular connectivity index (MCI) and valence molecular connectivity index (VMCI) can quantify and characterize the molecule’s structure [26]. Therefore, we plan to establish a method based on MCI and VMCI for predicting CMC to pre-select the modification schemes. Although CMCs have been predicted before, these methods were primarily designed for small molecular surfactants, making them unsuitable for Pluronic and its modification products [26,40]. Therefore, developing a novel method to accurately predict the CMC of polymer materials, such as F127, is necessary.

Our approach begun with calculating the MCI and VMCI of the raw materials, leveraging their known structures. Subsequently, we obtained the CMC of F127 and PCL-modified F127 using the ITC technique. The prediction model, established through a multivariate linear fit using the SPSS 17.0 software, demonstrates the precision of our method. Finally, we verified the accuracy of the predicting model by comparing the predicted values with the experimental values, further bolstering our confidence in its reliability.

##### Calculating the MCI and VMIC of Raw Materials

The *m*-th order MCI (where *m*-th means the order of connectivity index) is calculated using Equations (3) and (4).

(3) $$\delta_{i}=Z_{i}^{V}-h_{i}$$

In Equation (3), *δ_i_* means the connectivity degree; $Z_{i}^{V}$ is the number of valence electrons in the *i*-th atom; and *h_i_* is the number of hydrogen atoms on the *i*-th atom. For example, the *δ_i_* of methyl is 1, and the δ_i_ of methylene is 2.

(4) χkm=∑j=1nm∏i=1m+1(δi)j−0.5

In Equation (4), χkm means MCI; m denotes the order of the connectivity index; and k denotes the type of the fragment of the molecule, for instance, path (p), cluster (c), and path–cluster (pc). The types of fragments used in this work are shown in Figure S3. The number of fragments of order *m* is denoted by n_m_.

If the molecules contain heteroatoms, Equations (5) and (6) can be used to calculate the valence connectivity degree and VMCI [41].

(5) $$\delta_{i}^{V}=\frac{Z_{i}^{V}-h_{i}}{Z_{i}-Z_{i}^{V}-1}$$

In Equation (5), $\delta_{i}^{V}$ means the valence connectivity degree; $Z_{i}^{V}$ is the number of valence electrons in *i*-th atom; $Z_{i}$ denotes the number of all electrons in the *i*-th atom; and $h_{i}$ is the number of hydrogen atoms on *i*-th atom. For example, the $\delta_{i}^{V}$ of methyl is 1, and the $\delta_{i}^{V}$ of ether group is 6 (Figure S4).

(6) χkVm=∑j=1nm∏i=1m+1(δiV)j−0.5

In Equation (6), χkVm denotes VMCI; the order of the connectivity index is denoted by m; and k denotes the type of the fragment of the molecule, for instance, path (p), cluster (c), and path–cluster (pc). The number of fragments of order *m* is denoted by n_m_. In addition, the F127 was used as an example to demonstrate the MCI and VMCI calculation process in Supporting Information S2.

##### Testing the CMC of F127 and Modified F127 by ITC

ITC is a precise method for obtaining the heat change during the experiment. Approximately 10 mg of F127, F127-PCL2000, F127-PCL3500, F127-PLA2000, and F127-PLA3500 were carefully weighed and dissolved in 1 mL of normal saline using ultrasound. Following this, 40 μL of each solution was added to the syringe, and 200 μL of normal saline was added to the sample cell. The titration temperature was 40 °C, and the stirring speed was 750 rpm, with 2 μL per drop and one drop every 120 s. The heat change was recorded, and the peak area was calculated to draw the calorimetric curve using the Origin 8.0 software. The CMC of each material was precisely identified as the inflection point of the first-order derivative curve of its calorimetric curve.

##### Establishing the Mathematical Model for Predicting CMC

The MCI and VMCI of F127, PCL, PLA, PCL-modified F127, and PLA-modified F127 were calculated using Equations (4) and (6), respectively. The CMC values of F127, F127-PCL, and F127-PLA were measured by ITC and analyzed using the Origin 8.0 software. The mathematical model for predicting the CMC of modified F127 was established using SPSS 17.0 through multivariate linear fitting. To establish the most suitable formula for predicting CMC, the following three criteria were considered in the calculation: correlation coefficient (*r*), Fisher’s ratio (*F*), and standard error (*s*). The fitting result with the highest *F* and *r* values and the smallest *s* value was selected as the initial equation for further fitting. The regression process continued until there were no significant differences in the *r* values of each index after adding new variables. This meticulous process led to the establishment of the final equation.

##### Testing the Accuracy of the Prediction Mathematical Model

In this section, we verified the accuracy of the prediction model for CMC using F127-PLA, F127-PCL, and F127-PLGA. Firstly, the MCI and VMCI of PLA, PCL, and PLGA were calculated using Equations (4) and (6), respectively. Simultaneously, the predicted CMC of PLA-, PCL-, and PLGA-modified F127 were calculated using the prediction model established above. Secondly, the CMC values of F127-PLA, F127-PCL, and F127-PLGA were tested using ITC and calculated with Origin 8.0. Finally, the predicted and tested values were compared. This comparison is important as it determines whether there is a significant difference, which is the ultimate goal of our experiment.

#### 3.1.4. Predicting the ΔG of Modified F127

The ΔG can be used to judge the difficulty of the self-assembly process [42]. It aids in pre-selecting a suitable hydrophobic group for F127 modification. Once the CMC of modified F127 is obtained using the aforementioned method, the ΔG of the polymer during micellization can be calculated using Equations (7) and (8). The change in enthalpy (ΔH) between the micelle dilution concentration and micelle dissociation concentration can be utilized to calculate the micellization enthalpy. Then, the change in entropy (ΔS) can be calculated using Equation (9). Determining the influence of molecular weight (MW) and structure on micellization before modification is also beneficial for pre-selecting amphiphilic polymer design schemes.

(7) $$X_{\mathrm{cmc}}=n_{p}/\left(n_{p}+n_{w}\right)$$

X_cmc_ represents the molar ratio of polymer in the micelle solution. The unit of this physical quantity is dimensionless. n_p_ represents the moles of polymer, and n_w_ represents the moles of water, both with units of mol.

(8) $$\Delta G=\ln\;X_{\mathrm{cmc}}$$

ΔG represents the change in Gibbs free energy during micellization. The unit of ΔG is kJ/mol.

ΔS = (ΔH − ΔG)/T(9)

where ΔH represents the change in enthalpy during micellization, with unit of kJ/mol. T represents the Kelvin temperature. The unit of T is Kelvin (K). ΔS represents the change in entropy during micellization, with the unit of kJ/mol.

### 3.2. Confirming the Advantages of Modified Product Synthesized by the Pre-Selected Scheme in Preparing High-Quality Nano-Micelles

As described above, by predicting HLB, CMC, and ΔG, we can pre-select a suitable modification scheme to obtain polymers that may exhibit advantages when preparing high-quality nano-micelles. However, as this is merely a prediction, we must confirm it using pharmaceutical evaluation methods. This can effectively verify the guiding role of our procedure in pharmaceutical applications.

#### 3.2.1. Evaluating the Appearance of Micelles Prepared by the Polymer Synthesized Using the Pre-Selected Scheme

In addition to the thermodynamic parameters, we further confirmed the advantage of modified F127 in micellization. The micelles prepared using modified F127 were analyzed for particle size, zeta potential, and morphology. We employed DLS to detect the micelles’ particle size and zeta potential at room temperature and observed the morphology of micelles using TEM under an accelerating voltage of 100 kV.

#### 3.2.2. Evaluating the DL of Micelles Prepared by the Polymer Synthesized Using the Pre-Selected Scheme

Because one of the goals of hydrophobic modification is to increase the DL of F127 [43], we can determine which hydrophobic groups should be used in modification from a pharmaceutical perspective. Therefore, CUR was chosen as a hydrophobic drug to investigate which type of hydrophobic group was more suitable for modifying F127 to enhance DL.

F127, F127-PLGA2500, F127-PLGA4700, F127-PLA2000, F127-PLA3500, F127-PLA5000, F127-PCL2000, F127-PCL3500, and F127-PCL5300 were used as polymer materials to prepare CUR-loaded micelles. In this work, these micelles were prepared using the film hydration method as described in our previous research [32]. After that, HPLC with a diode array detector was utilized to detect the concentration of CUR in the sample. The parameters are as follows: mobile phase ratio: methanol–water = 75–25, column temperature: 30 °C, flow rate: 1 mL/mim, detection wavelength: 430 nm. The DL and EE were calculated using Equations (10) and (11), respectively.

DL = M_d_/(M_d_ + M_p_) × 100% (10)

DL denotes the drug loading. M_d_ denotes the mass of the drug in the micelle. M_p_ denotes the mass of the polymer. The units of M_d_ and M_p_ are milligrams.

EE = M_d_/M_t_ × 100% (11)

EE denotes the encapsulation efficiency. M_d_ denotes the drug’s mass in the micelle. M_t_ denotes the total mass of the drug input during preparation. The units of M_d_ and M_t_ are milligrams.

#### 3.2.3. Verifying the Compatibility of CUR with the Polymer Synthesized Using Pre-Selected Scheme

Equilibrium dialysis experiments have been employed to evaluate the intermolecular interaction between drugs and plasma proteins [44,45]. In this work, an equilibrium dialysis experiment was conducted to investigate the compatibility between CUR and modified F127.

The dialysis bag, meticulously prepared and treated, was cut into small sections, each approximately 4 cm in length. One end was tied with cotton, and 2 mL of a blank micelle solution (modified F127, 1 mg/mL) was added. A glass bead was placed inside the bag to keep it vertical in the buffer. Subsequently, the other end of the dialysis bag was also tied securely and suspended in an Eppendorf pipe (EP) containing 18 mL of PBS with 5% PEG-400. The concentration of CUR in the PBS was 0.05 mg/mL, and the fluid levels inside and outside the bag were carefully adjusted to be equal. After sealing, the system was allowed to stand at 20 °C for 24 h until drug diffusion equilibrium was achieved. The drug concentration inside the dialysis bag (total concentration, Ct) and outside the bag (free drug concentration, Cf) were measured separately. Equation (12) was utilized to calculate the binding rate of the drug–polymer.

The binding rate of the drug-polymer = (C_t_ − C_f_)/C_t_ × 100% (12)

#### 3.2.4. Verifying Whether the F127 Modified with the Pre-Selected Scheme Could Enhance the Stability of Micelles

Building on the previous prediction results regarding the micellar ΔG of hydrophobic modified F127, we designed a series of experiments to further investigate the advantage of the F127 modified with pre-selected hydrophobic group in micelles stabilization. To characterize the stability of micelles, we measured the particle size, polydispersity index (PDI), zeta potential, and DL over 30 days. The micelles were stored at 4 °C, and the particle size, PDI, and zeta potential were measured by DLS every 5 days. DL rates were measured by HPLC every five days.

#### 3.2.5. Confirming the Drug Release Rate of F127 Modified According to the Pre-Selected Scheme

The compatibility between polymers and drugs will affect not only the DL of the polymers but also the drug release rate [46]. Therefore, we used an accumulative release curve to evaluate the property of modified F127 as the drug carrier.

The release curves of CUR were investigated using a dialysis bag (MWCO 5000 Da) with a shaking rate of 100 rpm at 37 °C in PBS containing 0.5% SDS. As a control group, CUR was solubilized in DMSO. A total of 2 mL CUR micelles or control solution (equivalent to 0.15 mg CUR) were dialyzed against 18 mL release media. A 2 ml sample was withdrawn at pre-determined time intervals (0 h, 0.5 h, 1 h, 2 h, 4 h, 8 h, 12 h, 24 h, and 48 h) and replaced with a fresh medium of equal volume. HPLC was used to determine the concentration of CUR in the sample. The accumulated percentage of released CUR was calculated using Equations (13) and (14). The release experiments were performed in triplicate.

(13) $$M_{t}=C_{i}\;\times \;20\;\mathrm{mL}+\sum_{i=1}^{n-1}C_{i}\;\times \;2\;\mathrm{mL}$$

Accumulative release rate (%) = (M_t_/M_i_) × 100% (14)

where M_t_ represents the total amount of drug released at the interval time point; C_i_ (mg/mL) represents the CUR concentration in the medium at the interval time point; $\sum_{i=1}^{n-1}C_{i}$ × 2 mL represents the total amount of drug released until the *i*-th time point; and M_i_ represents the initial amount of CUR in the dialysis bag.

## 4. Results and Discussion

### 4.1. Establishing a Novel Method to Pre-Select Pluronic F127 Modification Scheme

#### 4.1.1. Confirming the Modification Product

**Figure 2:**
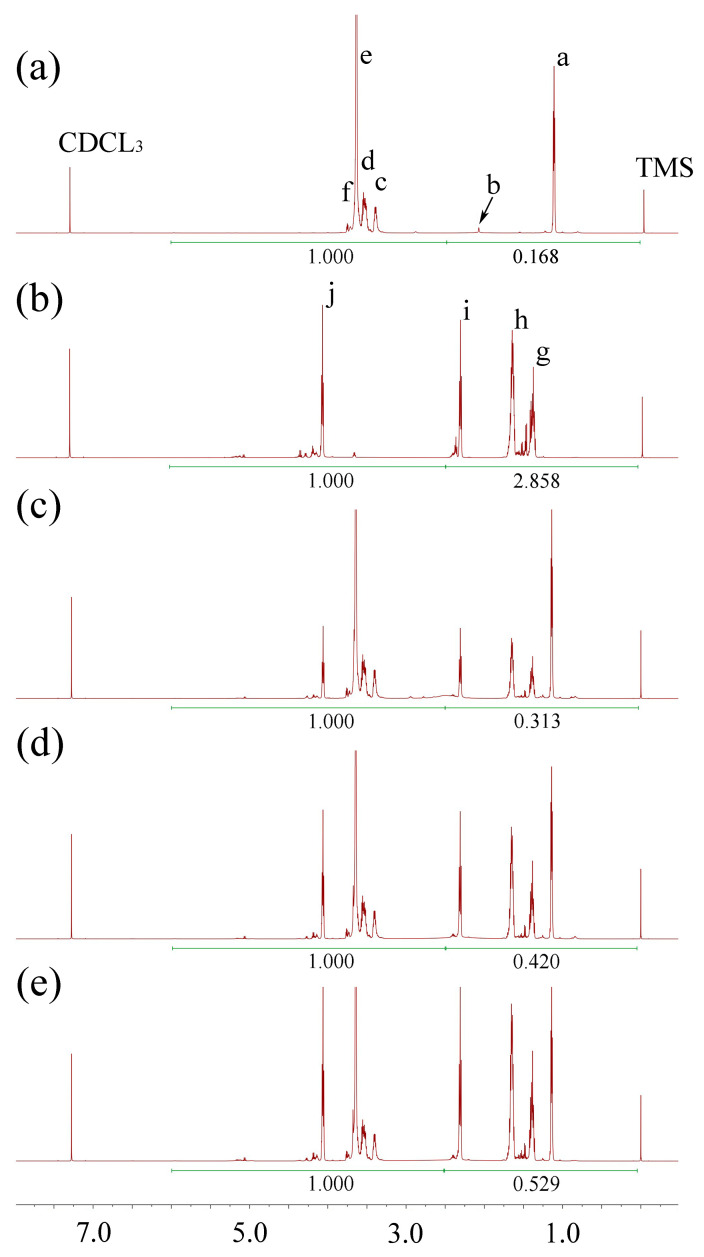
The ^1^H-NMR spectra of F127 and PCL-modified F127. (**a**) F127, (**b**) PCL, (**c**) F127-PCL2000, (**d**) F127-PCL3500, and (**e**) F127-PCL5300.

**Figure 3:**
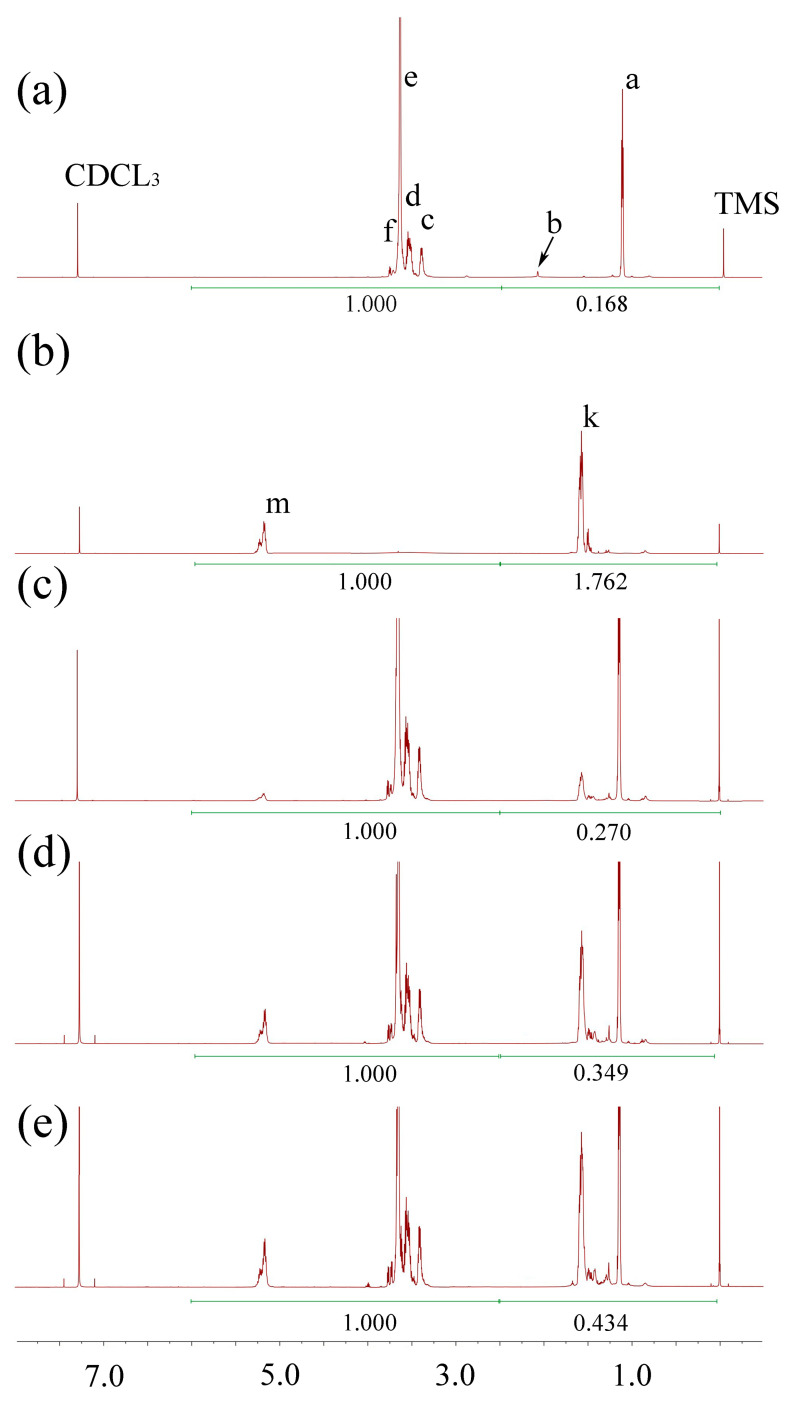
The ^1^H-NMR spectra of F127 and PLA-modified F127. (**a**) F127, (**b**) PLA, (**c**) F127-PLA2000, (**d**) F127-PLA3500, and (**e**) F127-PLA5000.

**Figure 4:**
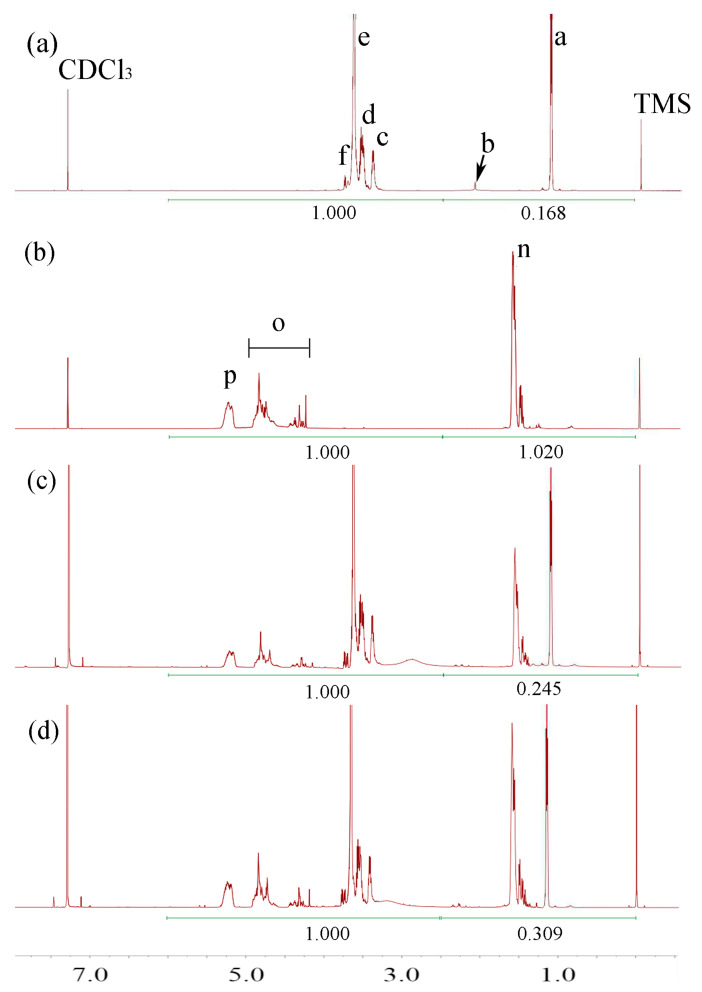
The ^1^H-NMR spectra of F127 and PLGA-modified F127. (**a**) F127, (**b**) PLGA, (**c**) F127-PLGA2500, and (**d**) F127-PLGA4700.

^1^H-NMR was used to confirm the result of the synthesis. Because there are no peaks from 7.35 ppm to 16.0 ppm, we only display the ^1^H-NMR spectra from −0.5 ppm to 7.5 ppm. Figure 2, Figure 3 and Figure 4 show the ^1^H-NMR of raw materials and modification products. The ^1^H-NMR showed the characteristic peaks of PCL, PLA, PLGA, and F127, which were also present in the spectra of modified F127. The presence of these characteristic peaks in the spectra of the modification product indicated a successful synthesis. Supporting Information S3 shows the details of the spectral analysis and structural confirmation.

Moreover, the molecular weight of modified F127 was obtained using MALDI-TOF-MS. The results are shown in Figures S5–S7. We used the Flex-Analysis Batch Process to calculate the average molecular weight, and the results are presented in Table S2. These results confirm that the modifications of F127 were successful. However, the molecular weight distribution was significantly widened after synthesis. The reasons for this may include two aspects: First, F127, PCL, PLA, and PLGA are not pure materials with a single molecular weight. Second, there is a random connection between the raw material molecules during the esterification process.

At the same time, we also measured the weight of the modification products. The results are shown in Table S3. On the one hand, compared to the input weight of F127, the weight of the modification products increased remarkably. On the other hand, compared to the theoretical collection weight of the products, the actual collection had a weight loss of about 10%. Given that the molecular weight difference and weight loss are about 10%, we speculate that the synthesis yield and purity of the products are about 90%.

In summary, we can confirm that the polymers were successfully synthesized and exhibit a relatively narrow molecular weight distribution, which could be utilized for further work.

#### 4.1.2. Predicting the HLB of Modified F127 Using ^1^H-NMR and Compound Formula

##### Drawing the Linear Curve of Surfactant HLB Value

After obtaining the NMR spectra for Span-60, Span-20, EL-35, Tween-80, Tween-20, and PEG-400, we utilized Equation (1) to calculate the R values. The results of each surfactant are shown in Table S4. The linear regression formula is presented in Equation (15). Figure S8 shows that the linear relationship between R and HLB is highly correlated (r = 0.999). Therefore, we can use Equation (15) to calculate the HLB of raw materials.

HLB = 18.962 × R + 2.212 (15)

##### Calculating the HLB of Raw Materials and Predicting the HLB of Modified F127

After obtaining the NMR spectra of F127, PCL, PLGA, and PLA, we calculated the R values according to Equation (1). Subsequently, we calculated the HLB values by substituting R into Equation (15). Table S5 shows the results, indicating that, for the same kind of materials, changes in MW do not significantly affect the HLB value. However, even with the same MW, the HLB of different kinds of hydrophobic groups exhibits remarkable differences. This suggests that the molecular structure has a greater influence on HLB than MW.

After the raw materials’ HLB were available, we could predict the HLB values of modified F127 using Equation (2). The results are shown in Table 1. When the ratio of the hydrophobic group increases in the entire molecules, the HLB value gradually decreases. Furthermore, the groups with stronger hydrophobicity have more impact on HLB, even though the hydrophobic groups have the same MW.

##### Testing the HLB of Modified F127 and Verifying the Accuracy of the Predicting Model

The ^1^H-NMR of modified F127 (Figure 2, Figure 3 and Figure 4) was processed using the method described above. The results, as depicted in Table 2, reveal a crucial correlation: for the same type of hydrophobic group, an increase in MW leads to a decrease in HLB, indicating that the polymers have stronger hydrophobicity; for different materials with the same MW, a more robust hydrophobicity group results in a smaller HLB value. These findings align with the HLB values of surfactants reported in pharmaceutical textbooks [15].

**Figure 5:**
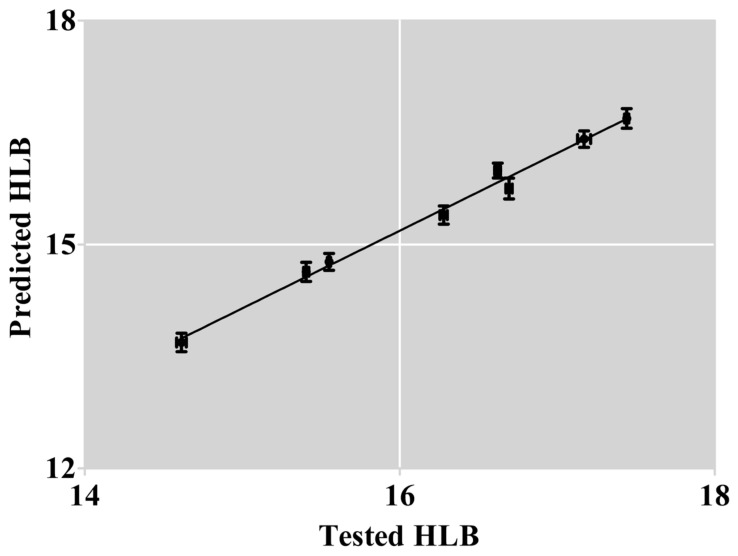
Scatter plot of the tested value versus the predicted value.

Figure 5 demonstrates the high accuracy (r = 0.996) of our prediction model in predicting the HLB of modified F127. This method, which relies on the ^1^H-NMR of raw materials and Equation (2), proved to be a reliable tool for pre-selecting modification schemes by accurately predicting the HLB values of the product.

HLB_p_ = 1.041 × HLB_t_ − 1.461 (16)

Equation (16) shows that the predicted values differ from the tested values. These differences could be attributed to the rough MW values of the polymers distributed in a particular range. Hence, some errors might be generated in the integral area of the hydrogen spectrum. Furthermore, dialysis removes molecules whose MW is lower than 10,000 Da. This MW distribution would influence the R value of modified F127 and have an impact on the tested HLB value.

To sum up, we achieved the aforementioned three goals:

1. Using the hydrogen spectrum to obtain the HLB value of raw materials;

2. Establishing a method to predict the HLB value of the target products;

3. Verifying the close relationship between the predicted and tested values.

Consequently, we can utilize this method to predict the HLB of modified products prior to the synthesis reactions.

#### 4.1.3. Predicting the CMC of Modified F127

As mentioned in the method, this section aims to find a simple equation for predicting the CMC of modified F127.

##### Calculating the MCI and VMIC of Raw Materials

Other previous reports used ten indices to search the relationship, which included five MCIs and five VMCIs, ranging from 0-th to 4-th order in each case [47]. We used Equations (3)–(6) to calculate the indices of F127, PLA, PLGA, and PCL. In Supporting Information S5, Table S6 lists all the MCIs.

##### Testing the CMC of F127 and Modified F127 by ITC

**Figure 6:**
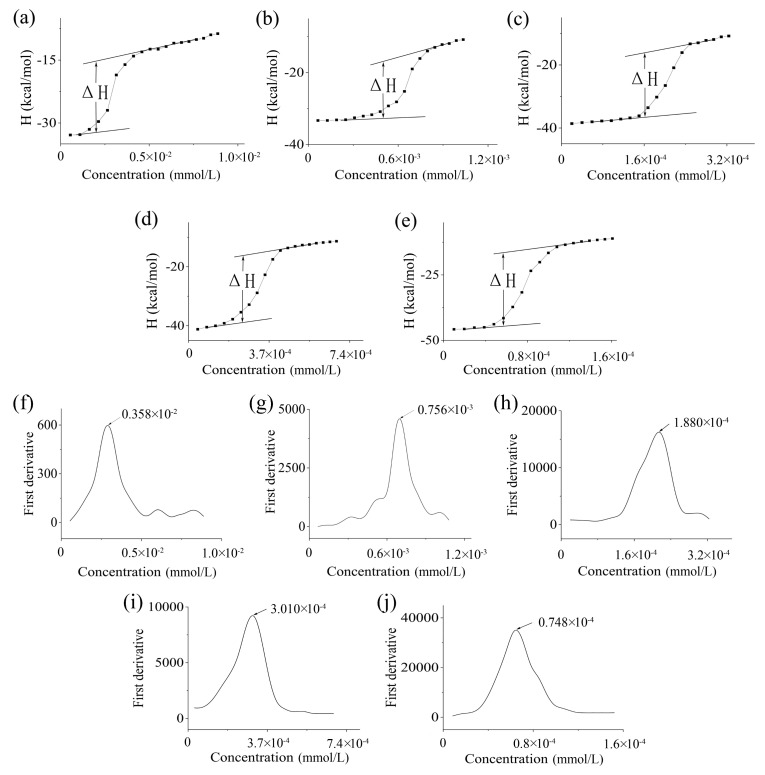
The enthalpy changes in titration of micellar F127 (**a**), F127-PLA2000 (**b**), F127-PLA3500 (**c**), F127-PCL2000 (**d**), and F127-PCL3500 (**e**) into normal saline at 40 °C. And the first derivative of the calorimetry curve of F127 (**f**), F127-PLA2000 (**g**), F127-PLA3500 (**h**), F127-PCL2000 (**i**), and F127-PCL3500 (**j**). ΔH denotes the enthalpy change in titration. The arrows in the figure point out the CMC of these polymers.

During the process of titration, ΔH were recorded. Consequently, we used Origin 8.0 to process the data, and the resulting curves are shown in Figure 6. As shown in Table 3, when we used PCL or PLA to modify F127, the CMCs were lower than that of F127. First, as the MW of hydrophobic groups increases, the CMC of the products modified with the same materials decreases. Second, when the MW does not show a remarkable difference, the CMC of the products modified by PCL is lower than that of those modified by PLA.

ITC, a method we employed, successfully obtained the CMC of modified F127. This method offers distinct advantages over surface tension, electric conductivity, light scattering, and fluorescence methods. First, it provides the CMC faster than other methods [28]. Second, it provides the CMC as well as the values of ΔH, ΔS, and ΔG [48], thereby enhancing the comprehensiveness of our findings.

##### Establishing the Mathematical Model for CMC Prediction

Table 4 displays the statistical results of the first step. The most suitable formula in this step is Equation (17), which contains χV1.

(17) lg CMC=−0.00846 × χV1+0.598

In further work, we added the remaining indices gradually. As the r values of the second step are presented in Table 4, adding other indices in the second step did not make a remarkable difference. Therefore, Equation (17) is the suitable formula for predicting CMC, which correlates with the molecular structure.

##### Verifying the Accuracy of the Prediction Model

**Figure 7:**
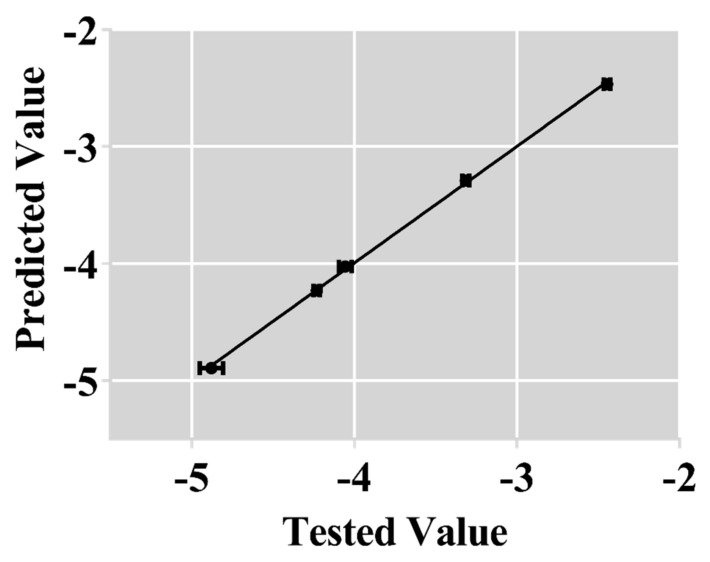
Scatter plot of the calculated values versus the tested values of lg CMC.

The predicted CMC values of modified F127 were calculated using Equation (14), and the results are shown in Table 5. In addition, Figure S9 and Table 5 display the tested CMCs of modified F127, obtained using ITC and Origin 8.0. As shown in Figure 7, the experimentally determined values correlate strongly with predicted values (r = 0.999). Hence, this method can accurately predict the CMC of modified F127.

#### 4.1.4. Predicting the ΔG in the Process of Micelle Formation of Modified F127

From the thermodynamic principle, we can predict the ΔG in the micellization before modification and explain why PCL is more suitable for F127 modification than PLA and PLGA.

The CMC was obtained from Equation (17). Then, we utilized Equations (7) and (8) to calculate the ΔG in micellization. By comparing and analyzing the data in Table 6, we observe that, when the polymer self-assembles to form micelles, ΔG is always negative, and the absolute value of ΔG increases with the increase in the MW of hydrophobic groups. Comparing our research data with the literature and analyzing it with the Second Law of Thermodynamics [17,41], we find that the micellization process becomes easier as the MWs of hydrophobic groups increase; moreover, it is a spontaneous process. The lower values of micellization ΔG indicate that F127-PCL molecules are thermodynamically easier to self-assemble into micelles than F127 and other modification products. Thus, this method proves an index value for pre-selecting hydrophobic groups before the modification.

In this method, we do not verify the accuracy of the ΔG-predicting model. However, Figure S10 demonstrates that, when we obtained the CMC, we also obtained a consistent ΔG. The accuracy of the CMC-predicting model was tested in the section entitled “Verifying the Accuracy of the Prediction Model”.

From the calorimetric curve of F127 and modified F127, we obtained the ΔH (Figure 6 and Figure S9). The enthalpy during the titration process was negative (an exothermic process) for all polymers considered in this paper. The possible contributions of such exothermic enthalpy profiles include the dilution and dissociation of micelles. Therefore, micelle formation is an endothermic process after considering these factors. We then calculated the ΔS in micellization using Equation (9). The ΔS of each group is a positive number, contributing to the negative ΔG.

Meanwhile, ΔS positively correlates with the MW and hydrophobicity of hydrophobic groups. Analyzing other relevant studies suggests that micellization is divided by entropy [49,50]. When ΔS is larger, the polymer is more likely to form micelles by self-assembly. In addition, all the ΔH and ΔS values in Table 6 are positive, illustrating that the major intermolecular interactions of F127 and modified F127 in the micellization are hydrophobic interactions [15]. These interactions are also consistent with the process by which hydrophobic groups of amphiphilic molecules self-assemble to form micelles in water. These results also indicate that PCL5300 is more suitable for F127 modification in our work.

Intending to select suitable polymer materials for micelle preparation, we chose PLGA, PLA, and PCL, which have similar MWs for F127 modification, and analyzed their ^1^H-NMR spectra and calorimetric curves. By establishing methods to predict HLB, CMC, and ΔG, we could determine which hydrophobic groups would contribute more to the micellization before modification. The results show that the molecular structure had a greater effect on micelle formation than MW. Using this prediction model, we selected PCL5300 to modify F127. The resulting HLB, CMC, and ΔG values became smaller, making the micellization process more accessible. This procedure could be a valuable tool for obtaining more suitable polymer materials for micelle preparation.

While our studies have provided preliminary prediction models for HLB, CMC, and ΔG, we acknowledge that we have only discussed the situation of chain segments. In our future studies, we plan to expand our scope to include polymers with benzene ring structures, which could be utilized to further verify the accuracy of our prediction models. We also note the existence of studies regarding the influence of benzene rings on surfactant and amphiphilic polymers [51]. It is generally believed that the benzene ring contributes little to hydrophobicity, a hypothesis that aligns with the chemical shifts of the benzene ring, which are higher than 2.5 ppm in the ^1^H-NMR.

### 4.2. It Is Confirmed That F127 Modified with the Pre-Selected Scheme Exhibits Advantages in Micelle Formation

#### 4.2.1. Using TEM and DLS to Confirm the Advantage of F127-PCL5300 in Micellar Morphology

**Figure 8:**
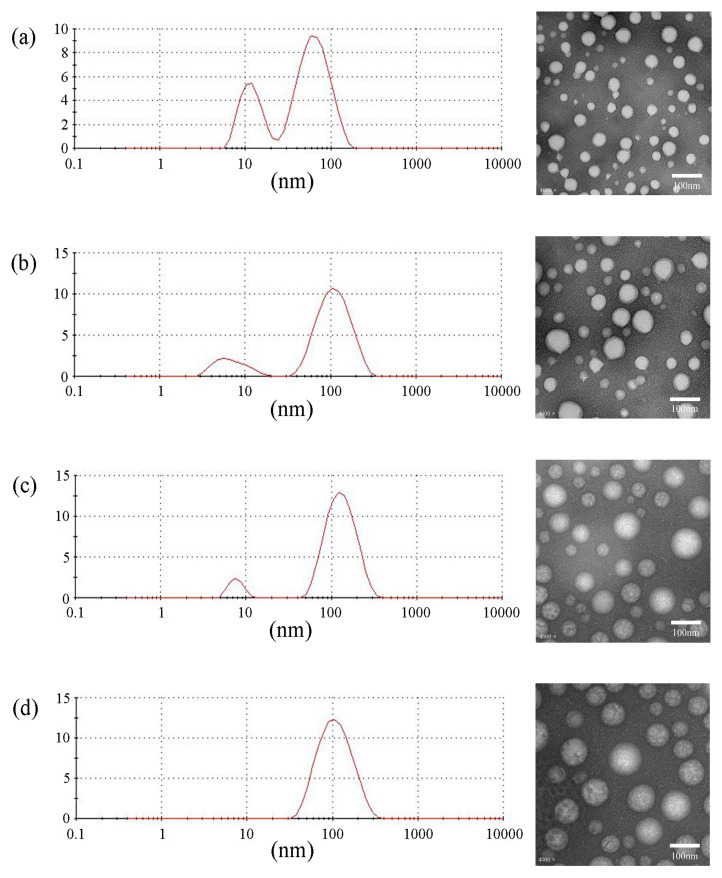
The DLS and TEM results of micelles. (**a**) F127, (**b**) F127-PLGA4700, (**c**) F127-PLA5000, (**d**) F127-PCL5300.

Moreover, we used F127, F127-PLGA4700, F127-PLA5000, and F127-PCL5300 as model materials for preparing micelles. These micelles were prepared using the film hydration method, as in our previous research [30,32]. After preparation, we used TEM and DLS to determine the particle size, PDI, and zeta potential. Figure 8 and Table 7 show that F127-PCL5300 is more suitable for forming homogeneous micelle particles than other modification products in the same preparation conditions. The hydrophobicity and molecular weight of the hydrophobic groups can affect the ΔG of modified products in the self-assembly process. This correlation can help us screen suitable hydrophobic groups for F127 modification. It also underscores the importance of pre-selecting hydrophobic groups for amphiphilic polymer design.

#### 4.2.2. PCL Modification Has Advantages in Increasing the Drug-Loading Rate of CUR

As shown in Table 8, if the focus is on increasing the DL of CUR, PCL5300 is more suitable for modifying F127. On the one hand, in these micelles, the DL of CUR gradually increases as the HLB of the polymer decreases. On the other hand, when the MWs of hydrophobic groups are similar, F127-PCL loads more CUR than F127-PLA and F127-PLGA; hence, hydrophobicity might play a key role here, as PCL exhibits the strongest hydrophobicity among the materials used in our work. Other previous studies on amphiphilic polymer synthesis support this conclusion, having drawn similar results, such as increasing the ratio of hydrophobic groups in amphiphilic polymer molecules enhances the DL of hydrophobic drugs [52].

#### 4.2.3. PCL Modification Offers More Advantages in Enhancing the Equilibrium Binding Ratio of CUR

As shown in Table 9, the equilibrium binding ratios of the drug–polymer increase with the decrease in the polymers’ HLB. This indicates that hydrophobic modification can enhance the intermolecular interaction between the drug and polymer, and an increase in the polymer’s hydrophobicity is conducive to carrying or binding more CUR. The DL results in Table 8 can mutually corroborate these equilibrium binding ratios, and the results of the two experiments are consistent with the relevant theories of surfactants and amphiphilic polymers in pharmaceutical textbook [15]. In this way, we can use the prediction HLB of the product to pre-select the suitable hydrophobic group for modifying F127.

The calorimetric curve depicting the intermolecular interaction between the drug and the polymer should be established to study the compatibility between them in further works. In this way, we can provide theoretical support for polymer synthesis from the perspective of micellar formation, as well as guidance for increasing micellar drug loading, which is more meaningful in pharmaceutical applications.

#### 4.2.4. The Micelles Prepared by F127-PCL Exhibited Better Stability

**Figure 9:**
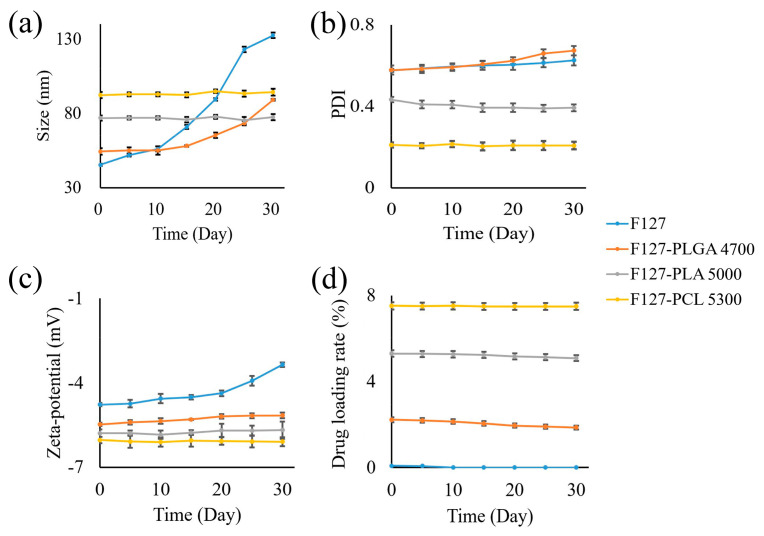
The change in particle size (**a**), PDI (**b**), zeta potential (**c**), and drug loading rate (**d**) of each micelle in 30 days.

**Figure 10:**
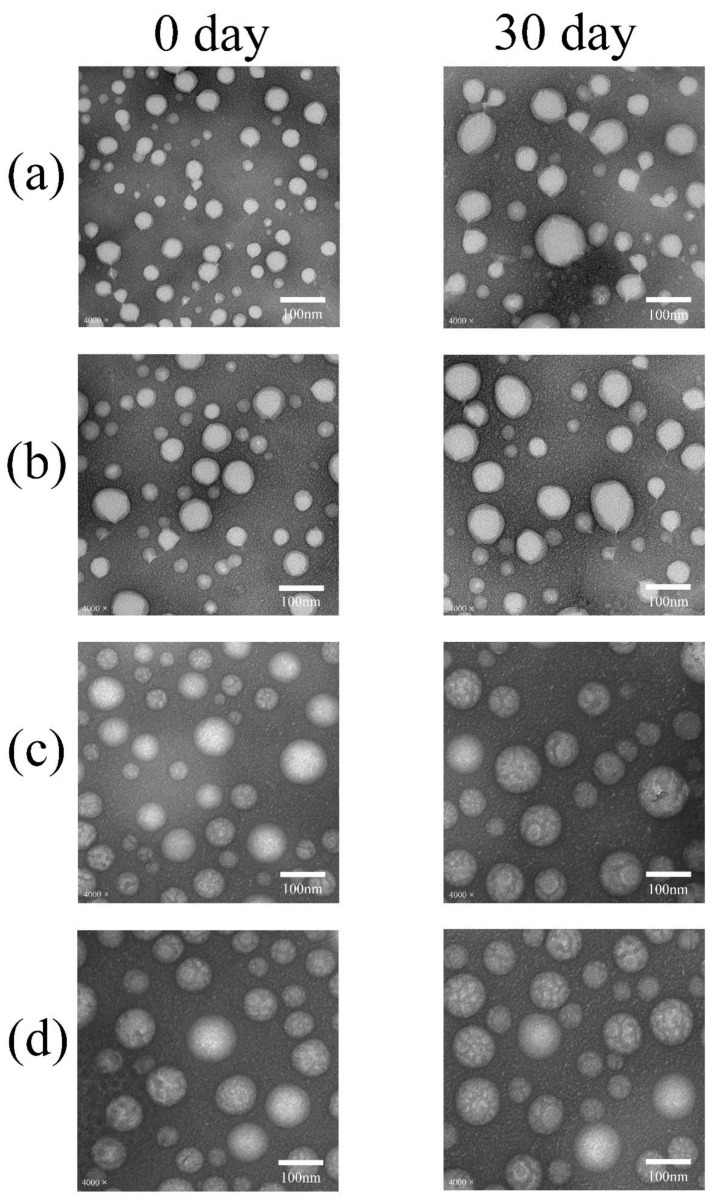
The appearance change in micelles in 30 days. (**a**) F127, (**b**) F127-PLGA4700, (**c**) F127-PLA5000, and (**d**) F127-PCL5300.

Figure 9 displays the particle size, PDI, zeta potential, and drug loading rate of each micelle over a period from 0 to 30 days. By comparing the changes in these four indexes, we can find that F127-PCL has a substantial advantage in micelle stability. For example, Figure 10 shows that the particle size of F127 micelles increases continuously within 0–30 days, while the particle sizes of F127-PLGA4700, F127-PLA5000, and F127-PCL5300 experience little or no significant change. When combined with the results for the other three polymers studied in this work, modification with PCL5300 was more conducive to forming stable amphiphilic polymer micelles. This is because the smaller ΔG associated with the micellization of F127-PCL (Table 6) indicates that the resulting preparation is closer to a thermodynamic equilibrium state, thus rendering it more stable [53,54].

#### 4.2.5. Hydrophobic Modification Slows Down the Release Rate of CUR in Micelles

After preparing drug-loaded micelles, we tested the samples and plotted the cumulative release curves of free CUR and eight CUR-loaded micelles. The experimental results presented in Figure S11 demonstrate that the release rate of the CUR gradually decreased with the increase in the polymer’s hydrophobicity. This phenomenon has also been reported in other studies [30,31]. However, this does not imply that increasing the polymer’s hydrophobicity is detrimental to the preparation of drug-loading micelles. The critical aspect lies in balancing the conflict between increasing the DL and accelerating the release rate. The current approach to resolving this contradiction involves using hydrazone, acetal, diselenide, disulfide bonds, and other degradable linkers to connect hydrophilic and hydrophobic groups, allowing the micelles to release the drugs quickly in response to these stimuli through micellar disintegration [55]. From another perspective, these results also confirm that PCL modification has advantages in enhancing intermolecular interactions. Additionally, some reports have shown that ionic compounds or hydrocarbon compounds can alter the interaction forces between the drug and the material, which can improve the stability and modulate the release rate [56,57].

## 5. Conclusions

Based on the existing experience and consensus, we developed a procedure for predicting the key properties of modified products. The aim of procedure is to pre-select a F127 modification scheme for preparing high-quality nano-micelles. This procedure establishes a connection between the structure of raw materials and the quality of micelles prepared using these products, through the evaluation of the HLB, CMC, and ΔG of the products. In this work, we pre-selected and obtained F127-PCL5300, which exhibits advantages in micelle formation, stability, DL of CUR, and the equilibrium binding ratio. It confirms that PCL5300 is a more suitable hydrophobic group for modifying F127 to prepare drug-loaded micelles. Compared to trial and error, this procedure helps us save time and costs in establishing drug-loading micelles. Furthermore, this pre-selection method presents a novel idea for the design and synthesis of amphiphilic polymers.

## Figures and Tables

**Table 1:** The predicted HLB of modified F127 ($\bar{X}$ ± S).

Group	MW of F127	HLB of F127	MW of Hydrophobic Group	HLB of Hydrophobic Group	Predicted HLB
F127-PLGA	14,600	18.434 ± 0.025	2500	11.596 ± 0.029	16.690 ± 0.131
			4700	11.580 ± 0.011	15.750 ± 0.139
F127-PLA			2000	9.038 ± 0.010	16.413 ± 0.111
			3500	9.055 ± 0.015	15.395 ± 0.121
			5000	9.086 ± 0.001	14.634 ± 0.128
F127-PCL			2000	7.076 ± 0.002	15.991 ± 0.099
			3500	7.122 ± 0.010	14.768 ± 0.113
			5300	7.154 ± 0.006	13.689 ± 0.124

**Note:** MW denotes molecular weight. HLB denotes hydrophile–lipophile balance.

**Table 2:** The tested HLB value of modified F127 ($\bar{X}$ ± S, n = 3).

Name	H_w_	Ho	R	HLB
F127-PLGA2500	1.000	0.245 ± 0.001	0.803 ± 0.001	17.441 ± 0.014
F127-PLGA4700	1.000	0.309 ± 0.002	0.764 ± 0.001	16.694 ± 0.020
F127-PLA2000	1.000	0.267 ± 0.003	0.789 ± 0.002	17.171 ± 0.041
F127-PLA3500	1.000	0.348 ± 0.002	0.742 ± 0.001	16.279 ± 0.023
F127-PLA5000	1.000	0.436 ± 0.002	0.696 ± 0.001	15.406 ± 0.017
F127-PCL2000	1.000	0.315 ± 0.002	0.76 ± 0.002	16.622 ± 0.018
F127-PCL3500	1.000	0.421 ± 0.002	0.704 ± 0.001	15.551 ± 0.010
F127-PCL5300	1.000	0.528 ± 0.004	0.654 ± 0.002	14.614 ± 0.031

**Note:** H_w_ denotes the relative coefficients of hydrophilic groups. H_o_ denotes the relative coefficients of hydrophobic groups. R presents the ratio of hydrophilic groups in the amphiphilic polymer.

**Table 3:** The CMC of F127 and its homologies ($\bar{X}$ ± S, n = 3).

Groups	CMC (mmol/L)
F127	0.358 × 10^−2^ ± 0.003 × 10^−2^
F127-PLA2000	0.756 × 10^−3^ ± 0.002 × 10^−3^
F127-PLA3500	0.188 × 10^−3^ ± 0.005 × 10^−3^
F127-PCL2000	0.301 × 10^−3^ ± 0.005 × 10^−3^
F127-PCL3500	0.748 × 10^−4^ ± 0.041 × 10^−4^

**Note:** CMC denotes the critical micelle concentration.

**Table 4:** The results derived by step-wise fitting.

Stage		^0^χ	^1^χ	^2^χ	^3^χ	^4^χ	^0^χ^v^	^1^χ^v^	^2^χ^v^	^3^χ^v^	^4^χ^v^
First step	r	0.956	0.985	0.950	0.469	0.433	0.974	**0.993**	0.990	0.363	0.402
	F	31.787	97.018	27.797	0.844	0.693	55.270	**221.885**	150.146	0.455	0.577
	s	0.217	0.128	0.230	0.652	0.665	0.168	**0.085**	0.103	0.688	0.676
Second step	r	0.526	0.577	0.546	0.528	0.593	0.567	**-**	0.522	0.543	0.509

**Note:** Bold font was used to show the important values; r denotes the correlation coefficient; F denotes the Fisher ratio value; s denotes the standard error; ^1^χ^v^ was used in Equation (14), and it was not used in the second step. In this table, it is labelled by “-”.

**Table 5:** The predicted lg CMC and tested lg CMC of F127, F127-PLGA2500, F127-PLGA4700, F127-PLA5000, and F127-PCL5300 (n = 3).

Groups	Predicted lg CMC	Tested CMC ($\bar{X}$ ± S)	Tested lg CMC ($\bar{X}$ ± S)
F127	−2.468	0.358 × 10^−2^ ± 0.003 × 10^−2^	−2.447 ± 0.003
F127-PLGA2500	−3.292	0.484 × 10^−3^ ± 0.004 × 10^−3^	−3.315 ± 0.003
F127-PLGA4700	−4.028	0.879 × 10^−4^ ± 0.029 × 10^−4^	−4.056 ± 0.014
F127-PLA5000	−4.231	0.586 × 10^−4^ ± 0.008 × 10^−4^	−4.231 ± 0.006
F127-PCL5300	−4.894	0.132 × 10^−4^ ± 0.010 × 10^−4^	−4.879 ± 0.033

Note: CMC denotes the critical micelle concentration. The unit of CMC is mmol/L.

**Table 6:** The thermodynamic parameters of the micellization of F127 and modified F127 ($\bar{X}$ ± S, n = 3).

Groups	ΔH (kJ/mol)	CMC (mmol/L)	ΔG (kJ/mol)	ΔS (kJ/mol·K)
F127	77.684 ± 1.712	0.358 × 10^−2^ ± 0.003 × 10^−2^	−43.112 ± 0.020	0.386 ± 0.006
F127-PLGA2500	78.474 ± 1.783	0.484 × 10^−3^ ± 0.004 × 10^−3^	−48.315 ± 0.020	0.405 ± 0.005
F127-PLGA4700	115.254 ± 0.767	0.879 × 10^−4^ ± 0.029 × 10^−4^	−52.755 ± 0.087	0.537 ± 0.003
F127-PLA2000	82.986 ± 3.024	0.756 × 10^−3^ ± 0.002 × 10^−3^	−47.156 ± 0.065	0.416 ± 0.010
F127-PLA3500	96.076 ± 2.128	0.188 × 10^−3^ ± 0.005 × 10^−3^	−50.771 ± 0.067	0.469 ± 0.007
F127-PLA5000	123.67 ± 4.453	0.588 × 10^−4^ ± 0.009 × 10^−4^	−53.803 ± 0.037	0.567 ± 0.014
F127-PCL2000	111.376 ± 2.322	0.301 × 10^−3^ ± 0.005 × 10^−3^	−49.552 ± 0.044	0.514 ± 0.008
F127-PCL3500	129.151 ± 2.957	0.748 × 10^−4^ ± 0.041 × 10^−4^	−53.177 ± 0.153	0.582 ± 0.009
F127-PCL5300	141.382 ± 2.836	0.132 × 10^−4^ ± 0.010 × 10^−4^	−57.684 ± 0.195	0.636 ± 0.008

Note: ΔH denotes the enthalpy change in micellization, ΔS denotes the entropy change in micellization, ΔG denotes the Gibbs free energy change in micellization, and CMC denotes the critical micelle concentration.

**Table 7:** The particle size, PDI, and zeta potential of micelles ($\bar{X}$ ± S, n = 3).

Groups	Particle Size (nm)	PDI	Zeta Potential (mV)
F127	45.373 ± 1.52	0.575 ± 0.052	−4.765 ± 0.275
F127-PLGA4700	54.307 ± 2.014	0.577 ± 0.037	−5.478 ± 0.116
F127-PLA5000	76.682 ± 1.024	0.437 ± 0.016	−5.801 ± 0.133
F127-PCL5300	92.189 ± 1.368	0.207 ± 0.006	−6.007 ± 0.211

**Table 8:** Drug-loading rates of CUR in micelles prepared with F127 and its modification products ($\bar{X}$ ± S, n = 3).

Groups	EE (%)	DL (%)	HLB
F127	0.734 ± 0.005	0.072 ± 0.002	18.402 ± 0.010
F127-PLGA2500	14.809 ± 0.254	1.463 ± 0.016	17.441 ± 0.014
F127-PLGA4700	22.478 ± 0.329	2.200 ± 0.020	16.694 ± 0.020
F127-PLA2000	17.413 ± 0.283	1.690 ± 0.017	17.171 ± 0.041
F127-PLA3500	28.846 ± 0.689	2.773 ± 0.026	16.279 ± 0.023
F127-PLA5000	55.977 ± 0.799	5.229 ± 0.046	15.406 ± 0.017
F127-PCL2000	22.485 ± 0.275	2.173 ± 0.018	16.622 ± 0.018
F127-PCL3500	51.844 ± 0.468	4.873 ± 0.047	15.551 ± 0.010
F127-PCL5300	81.784 ± 1.553	7.519 ± 0.088	14.614 ± 0.031

**Note:** EE denotes the encapsulation efficiency, DL denotes drug loading, and HLB denotes hydrophile–lipophile balance.

**Table 9:** The equilibrium binding rate of F127 and modified F127 at 20 °C ($\bar{X}$ ± S, n = 3).

Groups	C_t_ (mg/mL)	C_f_ (mg/mL)	Equilibrium Binding Rate (%)	HLB
F127	0.0490 ± 0.0003	0.0483 ± 0.0003	1.426 ± 0.202	18.402 ± 0.010
F127-PLGA2500	0.0487 ± 0.0005	0.0469 ± 0.0005	3.696 ± 0.042	17.441 ± 0.014
F127-PLGA4700	0.0486 ± 0.0003	0.0448 ± 0.0002	7.882 ± 0.087	16.694 ± 0.020
F127-PLA2000	0.0485 ± 0.0004	0.0455 ± 0.0004	6.014 ± 0.077	17.171 ± 0.041
F127-PLA3500	0.0486 ± 0.0004	0.0440 ± 0.0003	9.527 ± 0.210	16.279 ± 0.023
F127-PLA5000	0.0487 ± 0.0002	0.0428 ± 0.0002	12.038 ± 0.103	15.406 ± 0.017
F127-PCL2000	0.0487 ± 0.0007	0.0446 ± 0.0006	8.543 ± 0.118	16.622 ± 0.018
F127-PCL3500	0.0486 ± 0.0004	0.0430 ± 0.0004	11.643 ± 0.303	15.551 ± 0.010
F127-PCL5300	0.0484 ± 0.0005	0.0415 ± 0.0005	14.178 ± 0.170	14.614 ± 0.031

**Note:** HLB denotes hydrophile–lipophile balance.

## Data Availability

The original contributions presented in this study are included in the article/Supplementary Materials. Further inquiries can be directed to the corresponding author.

## Supplementary Materials

The following supporting information can be downloaded at: https://www.mdpi.com/article/10.3390/polym17030349/s1. S1. Confirmation of the purification effect of F127 and demonstration of the steps of F127 modification. S2. Demonstration of the method of MCI calculation. S3. Confirmation of the structure of the modified F127. S4. Linear curve of surfactant HLB value and calculation the HLB of raw materials. S5. The data and figure for establishing the CMC, ΔG prediction model, and accumulative release curves.
