# Supplementary material for: Predicting the Key Properties of a Modified Product to Pre-Select a Pluronic F127 Modification Scheme for Preparing High-Quality Nano-Micelles
Source: Polymers (Basel). 2025 Jan 27;17(3):349. doi: 10.3390/polym17030349 (PMC11821254; doi:10.3390/polym17030349)
Supplement: Supplementary file 1 [file polymers-17-00349-s001.zip › polymers-3411295-supplementary.pdf]

Predicting the Key Properties of a Modified Product to Pre-Select a Pluronic F127 Modification Scheme for Preparing High-Quality Nano-Micelles

Jizheng Song [1,†], Yu Hu [1,†], Shiyu Yang [1], Dexue Liu [1], Yiider Tseng [2] and Lingjun Li [1,*]

[1] College of Pharmacy, Shandong University of Traditional Chinese Medicine, Jinan 250355, China
[2] Innovative Institute of Chinese Medicine and Pharmacy, Shandong University of Traditional Chinese Medicine, Jinan 250355, China
[*] Correspondence: 60030053@sdutcm.edu.cn
[†] These authors contributed equally to this work.

Supporting information 1

1 The Purifying Process of F127

2 g of F127 is added to a 250 ml Erlenmeyer flask and then dissolved with 200 ml of water. After that, 10 g of Cyano-modified silica is added to the solution and gently stirred for 16 h at 4 ℃. After 16 h, the silica materials are filtered out. To completely desorb the adsorbed F127 from the silica, 200 mL of methanol at 60 ℃ is used. The solution is then separated from the silica by filtration and dried using a rotary evaporator. After further drying the F127 in a vacuum at room temperature for 12h, we obtain the purified F127.

2 Confirming the Purification Effect of F127

In brief, the molecular weight distribution of impure and purified F127 was detected by SEC (Viscotek TDAmax, USA). Two columns in series (Agilent ZORMAX GF-250, diameter: 9.4 mm, length: 250 mm, particle size: 4 μm, USA) were used for analysis experiments. The mobile phase was methanol: water = 60: 40, the flow rate was set to 1.0 ml/min, the temperature was set at 40 ℃, the concentration was 0.5mg/ml, and the volume of each injection was 20 μl. The results of SEC are showed in Figure S1 and Table S1.

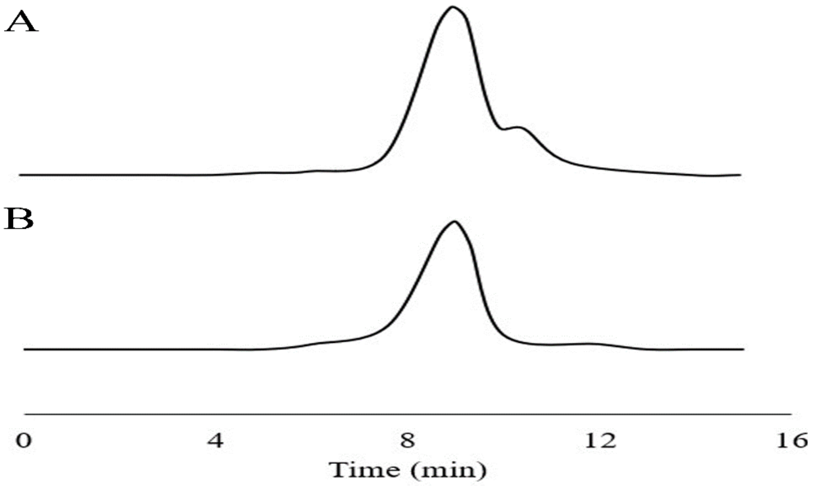

Figure S1. The SEC profiles of impurified F127 (A) and purified F127(B)

Table S1 The SEC results of impurified and purified F127 ($\bar{x}$±S, n=3)

Groups	M_n	Mw	PDI
Impurified F127	9,165.0±46.6	12,863.7±68.3	1.404±0.007
Purified F127	11,160.3±76.1	14,251.7±110.5	1.277±0.004

The MALDI-TOF-MS method meticulously detected the molecular weight distribution of both

impure and purified F127, effectively demonstrating the purification effect of F127. All samples were analyzed using an MALDI-TOF-MS instrument equipped with a 337nm nitrogen laser. Spectra were obtained in positive ion model with an acceleration voltage of 20 kV. CAHC (50mg/ml in DMF) was used as the matrix, and samples were dissolved in DMF (10mg/ml). They were mixed with a ratio of matrix: sample = 3: 1, and 0.6 μl of the mixed solution was meticulously spotted onto a MALDI sample plate. The results of MALDI-TOF-MS analysis are shown in Figure S2.

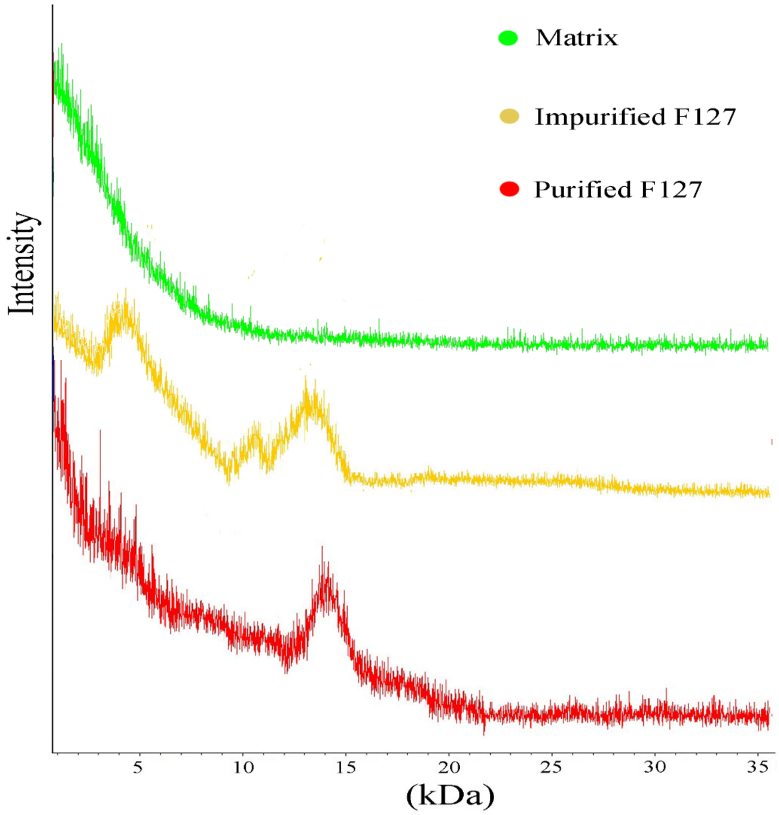

Figure S2. The mass spectra of matrix, impurified F127 and purified F127.

3 Modification of F127

In this work, we used an esterification reaction to synthesize all the polymer materials. First, PCL, PLA, or PLGA (0.3 mmol) were added to 3 Shrek bottles, respectively. Afterwards, F127 (0.1 mmol), DMAP (0.3 mmol), EDC (0.3 mmol) and TEA (0.3 mmol) were dissolved in 10 mL of dry dichloromethane (DCM) and then added to the Shrek bottle. In each bottle, the solution was stirred at room temperature for 48 h. Subsequently, rotary evaporation was used to remove DCM from the solutions, and the residues were dissolved in acetone. The solution were then placed into dialysis bag (molecular weight cut off (MWCO): 3,500 Da) and dialyzed against purified water to remove unreacted materials. Finally, PCL-, PLA-, and PLGA-modified F127 were obtained by freeze-drying.

Supporting information 2

Demonstrating the Method of MCI Calculation

${}^{m}\chi_{k}$ means MCI; the order of the connectivity index is denoted by m; k denoted the type of the fragment of the molecule, for instance, path (p), cluster (c), and path-cluster (pc). The types of fragments used in this work are shown in Figure S3.

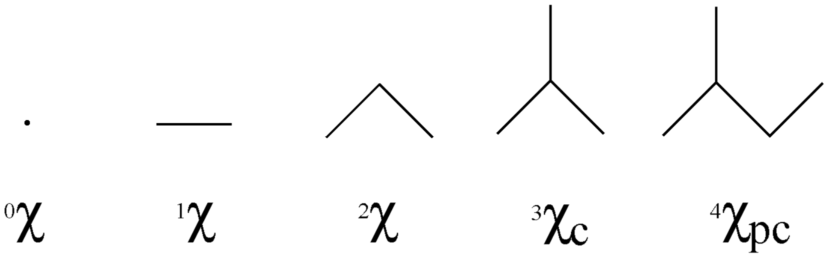

Figure S3 The fragments which were used to calculate the MCI.

We use F127 as an example to demonstrate the calculation process of the MCI. Figure S4 presents the δ_i and δ_i^V of each atom in the F127 molecule.

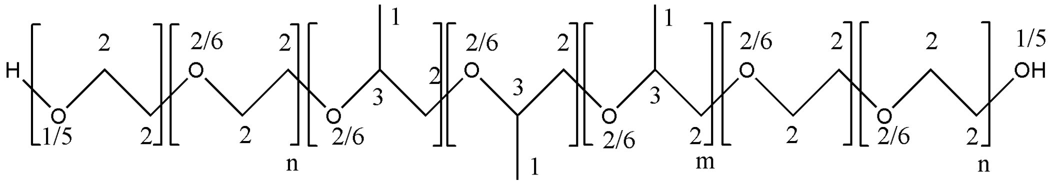

Figure S4. The δ_i and δ_i^V of each atom in F127 molecular. 1/5 denotes the oxygen atom's δ_i is 1 and δ_i^V is 5.

${}^{0}\chi=(2^{-0.5}+2^{-0.5}+2^{-0.5})\times232-2^{-0.5}+1^{-0.5}+1^{-0.5}+(2^{-0.5}+3^{-0.5}+1^{-0.5}+2^{-0.5})\times76=720.798$

${}^{1}\chi=((2\times2)^{-0.5}+(2\times2)^{-0.5})\times232+((2\times2)^{-0.5})\times230-(2\times2)^{-0.5}+(1\times2)^{-0.5}+(2\times1)^{-0.5}+((2\times3)^{-0.5}+(3\times1)^{-0.5}+(3\times2)^{-0.5})\times76+((2\times2)^{-0.5})\times75+((2\times2)^{-0.5})\times2=492.347$

${}^{2}\chi=((2\times2\times2)^{-0.5})\times232+((2\times2\times2)^{-0.5}+(2\times2\times2)^{-0.5})\times230-(2\times2\times2)^{-0.5}+(1\times2\times2)^{-0.5}+(2\times2\times1)^{-0.5}+((2\times3\times1)^{-0.5}+(2\times3\times2)^{-0.5}+(1\times3\times2)^{-0.5})\times76+((3\times2\times2)^{-0.5}+(2\times2\times3)^{-0.5})\times75+((2\times2\times2)^{-0.5}+(2\times3\times2)^{-0.5})\times2=373.884$

${}^{3}\chi=((2\times3\times1\times2)^{-0.5})\times76=21.939$

${}^{4}\chi=((2\times2\times3\times1\times2)^{-0.5})\times76+((2\times3\times1\times2\times2)^{-0.5})\times76=31.435$

${}^{0}\chi^{v}=(6^{-0.5}+2^{-0.5}+2^{-0.5})\times232-6^{-0.5}+5^{-0.5}+5^{-0.5}+(6^{-0.5}+3^{-0.5}+1^{-0.5}+2^{-0.5})\times76=627.943$

${}^{1}\chi^{v}=((6\times2)^{-0.5}+(2\times2)^{-0.5})\times232+((2\times6)^{-0.5})\times230-(6\times2)^{-0.5}+(5\times2)^{-0.5}+(2\times5)^{-0.5}+((6\times3)^{-0.5}+(3\times1)^{-0.5}+(3\times2)^{-0.5})\times76+((2\times6)^{-0.5})\times75+((2\times6)^{-0.5})\times2=362.449$

${}^{2}\chi^{v}=((6\times2\times2)^{-0.5})\times232+((2\times2\times6)^{-0.5}+(2\times6\times2)^{-0.5})\times230-(6\times2\times2)^{-0.5}+(5\times2\times2)^{-0.5}+(2\times2\times5)^{-0.5}+((6\times3\times1)^{-}$

$^{0.5}+(6\times3\times2)^{-0.5}+(1\times3\times2)^{-0.5})\times76+((3\times2\times6)^{-0.5}+(2\times6\times3)^{-0.5})\times75+((2\times2\times6)^{-0.5}+(2\times6\times3)^{-0.5})\times2=228.845$

$^{3}\chi^{v}=((6\times3\times1\times2)^{-0.5})\times76=12.667$

$^{4}\chi^{v}=((2\times6\times3\times1\times2)^{-0.5})\times76+((6\times3\times1\times2\times2)^{-0.5})\times76=14.128$

Supporting information 3

1 The ^{1}H-NMR of F127 and Its Hydrophobic Group Modifications

^{1}H-NMR was used to display the structures of raw materials and modification products. Figures 2, 3, and 4 show the ^{1}H-NMR of raw materials and modification products.

Firstly, in Figure 2, the ^{1}H-NMR dispalyed the characteristic peaks of PCL (g (δ=1.29 ppm), h (δ=1.57 ppm), i (δ=2.25 ppm) and j (δ=4.08 ppm)) and the peaks of F127 (a (δ=1.37 ppm), c (δ=3.38 ppm), d (δ=3.56 ppm) for polypropylene oxide, and the e (δ=3.64 ppm) and f (δ=3.77 ppm) for polyethene oxide) appeared in the ^{1}H-NMR of PCL-modified F127. Peak b (δ=2.00 ppm) should be noted, which belongs to the hydroxyl group at the end of F127. This hydroxyl group was consumed during the esterification of F127 and PCL. Therefore, peak b disappeared in the spectra of F127-PCL. These results could preliminarily confirm that the PCL modification was successful.

Secondly, in Figure 3, ^{1}H-NMR showed the characteristic peaks of PLA (k (δ=1.57 ppm) and m (δ=5.17 ppm)) and the peaks of F127 (a (δ=1.37 ppm), c (δ=3.38 ppm), d (δ=3.56 ppm), e (δ=3.64 ppm) and f (δ=3.77 ppm)) appeared in the ^{1}H-NMR of PLA modified F127. Peak b (δ=2.00 ppm) should be noted, which belongs to the hydroxyl group at the end of F127. This hydroxyl group was consumed during the esterification of F127 and PLA. Therefore, peak b disappeared in the spectra of F127-PLA. These results could preliminarily confirm that the PLA modification was successful.

Thirdly, in Figure 4, ^{1}H-NMR showed the characteristic peaks of PLGA (n (δ=1.61 ppm), o (δ=4.25-5.00 ppm), and p (δ=5.23 ppm)) and the peaks of F127 (a (δ=1.37 ppm), c (δ=3.38 ppm), d (δ=3.56 ppm), e (δ=3.64 ppm) and f (δ=3.77 ppm)) appeared in the ^{1}H-NMR of PLGA modified F127. Peak b (δ=2.00 ppm) should be noted, which belongs to the hydroxyl group at the end of F127. This hydroxyl group was consumed during the esterification of F127 and PLGA. Therefore, peak b disappeared in the spectra of F127-PLGA. These results could preliminarily confirm that the PLGA modification was successful.

In Figures 2, 3, and 4, as the molecular weight of the hydrophobic group increased, the integral area of the peak corresponding to PCL, PLA, and PLGA also increased. It was preliminarily indicated that we obtained the modified products with different molecular weights.

At the same time, the relative coefficients of H_o and H_w are shown in these figures, which were obtained by diving ∑Ao and ∑Aw by ∑Aw, respectively.

2 The MALDI-TOF Spectrum of F127 and Its Hydrophobic Group Modification Products

MALDI-TOF-MS confirmed molecular weights of the obtained products, the results are shown as

Figure S5, S6, and S7.

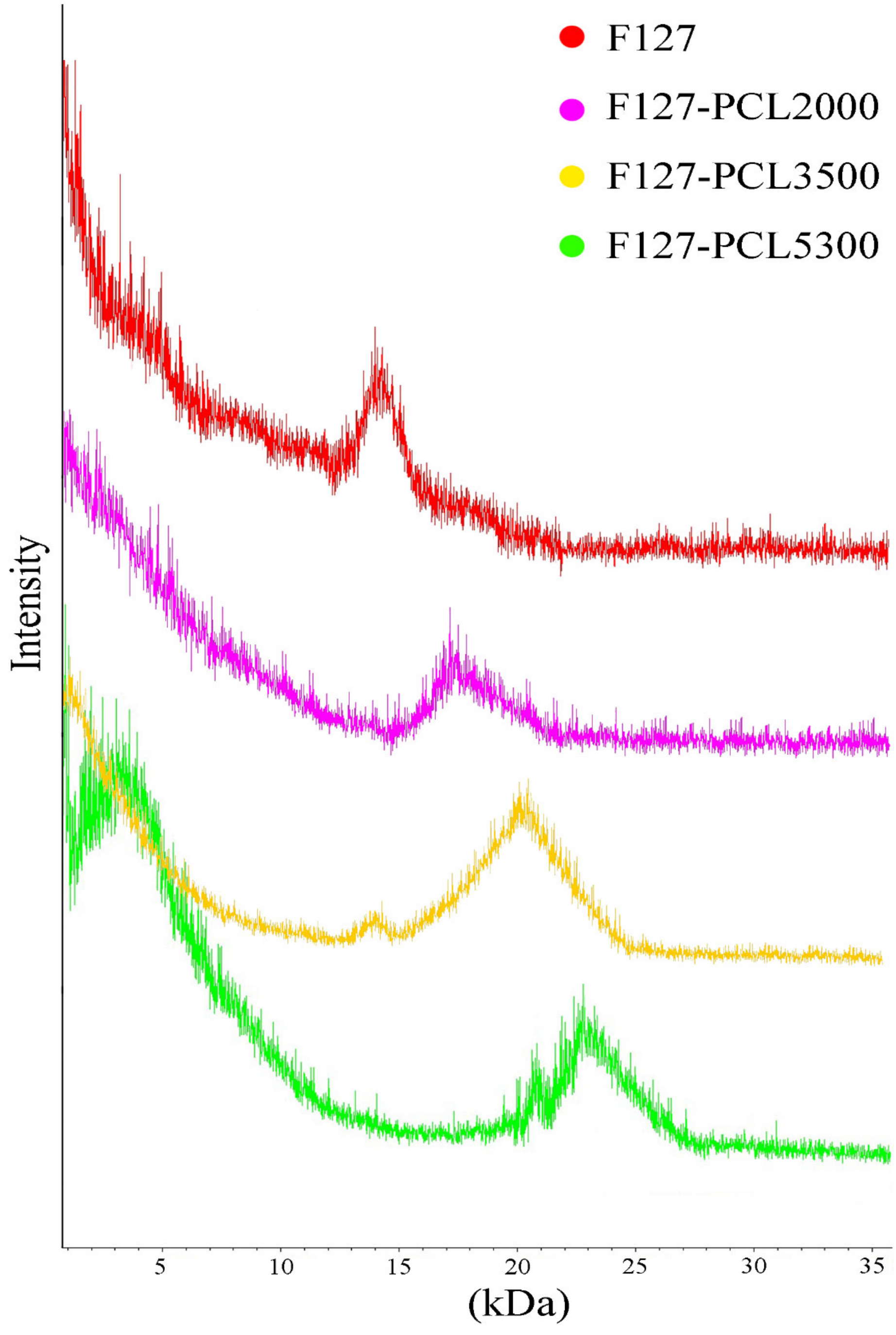

Figure S5. The mass spectra of F127 and PCL-modified F127.

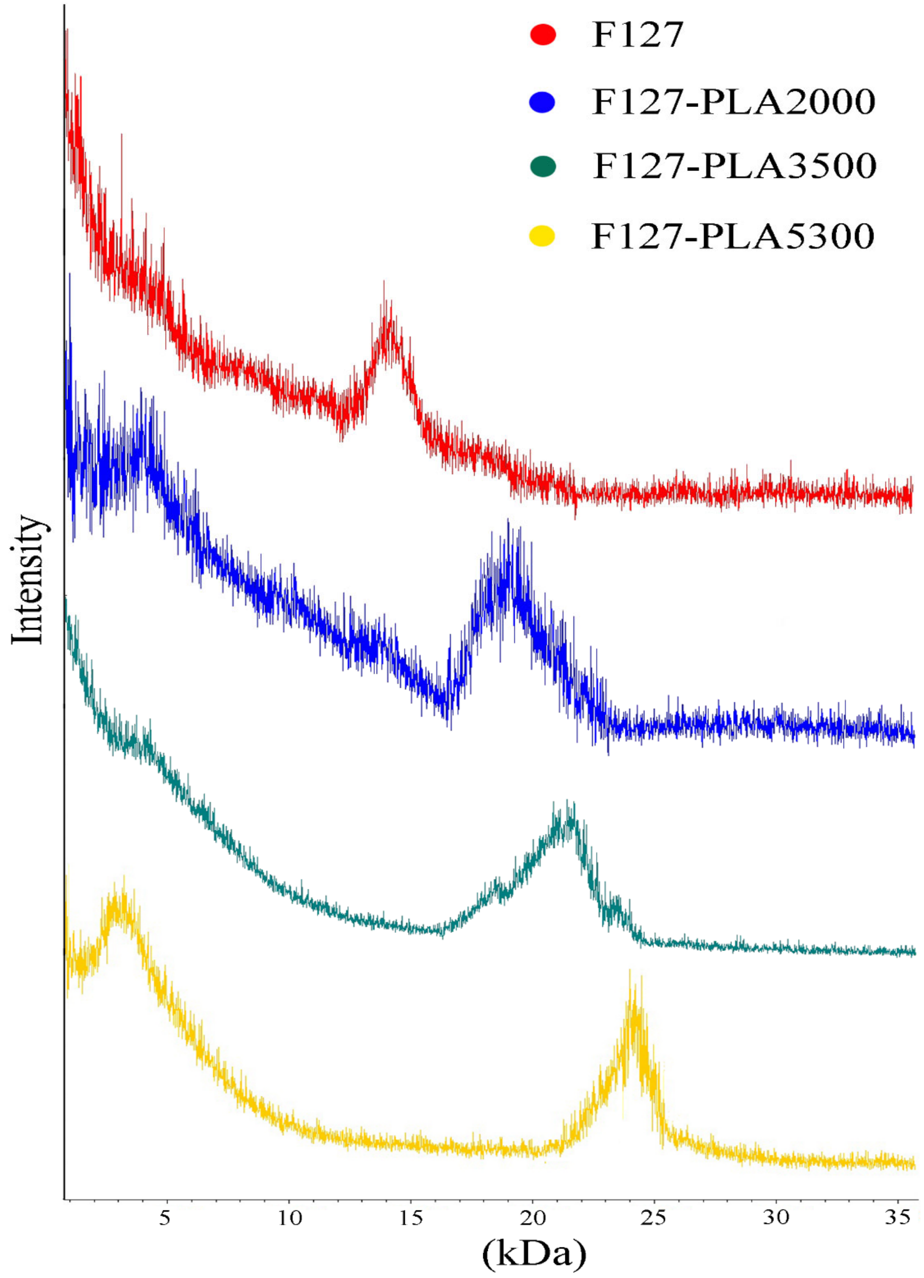

Figure S6. The mass spectra of F127 and PLA-modified F127.

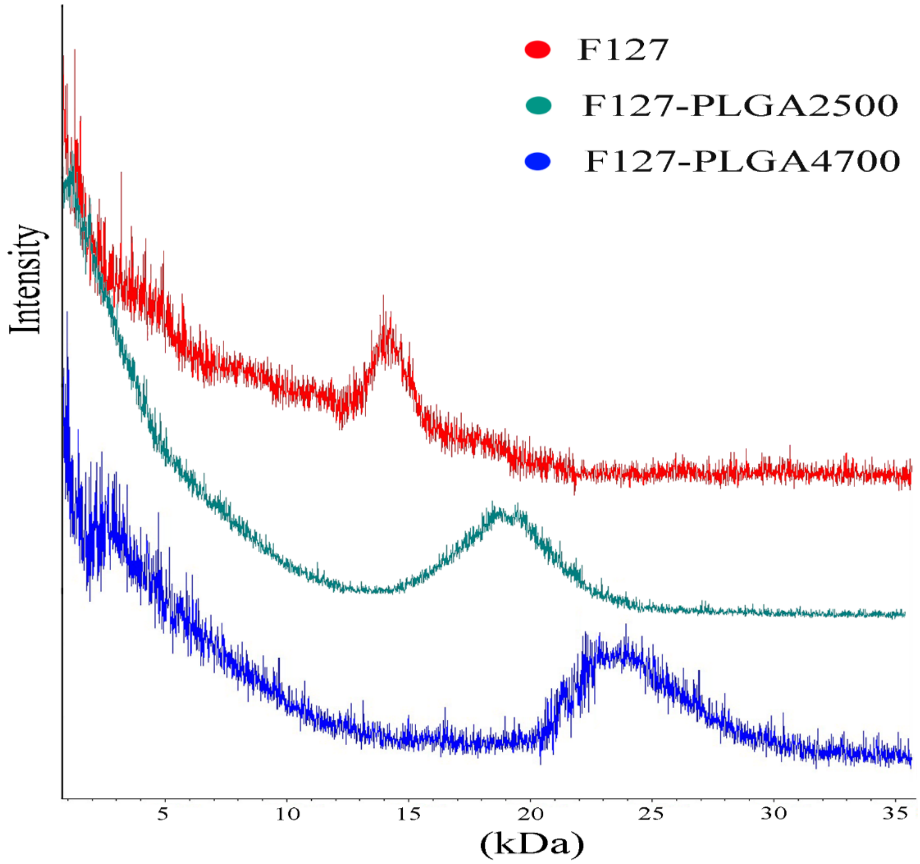

Figure S7. The mass spectra of F127 and PLGA-modified F127.

Table S2

The average molecular weight of F127 and its modification products (n=3)

Groups	Average molecular weight (Theoretical value)	Average molecular weight (Measured value) ($\bar{x}\pm S$)	Different value (%)
F127	14,600	14,114±66	3.40±0.40
F127-PLGA2500	19,564	17,486±96	11.4±0.83
F127-PLGA4700	23,964	21,688±280	10.4±1.31
F127-PLA2000	18,564	16,775±101	10.2±0.69
F127-PLA3500	21,564	19,723±98	8.99±0.58
F127-PLA5000	24,564	22,572±299	8.46±1.26
F127-PCL2000	18,564	17,371±145	6.67±0.82
F127-PCL3500	21,564	19,851±78	8.26±0.28
F127-PCL5300	25,164	22,593±300	11.43±1.19

Table S3

The weight of input materials and modification products (n=3)

Groups	Input material		Modification products		
	F127 (mg)	Hydrophobic group (mg)	Theoretical value (mg)	Measured value ($\bar{x}\pm S$) (mg)	Weight loss ($\bar{x}\pm S$) (%)
F127	1,460	-	-	-	-
F127-PLGA2500	1,460	750	1,956	1,780±22	9.22±1.13
F127-PLGA4700	1,460	1,410	2,396	2,203±89	10.46±1.40
F127-PLA2000	1,460	600	1,856	1,683±48	9.52±2.59
F127-PLA3500	1,460	1,050	2,156	1,989±109	9.48±2.71
F127-PLA5000	1,460	1,500	2,456	2,218±61	9.85±2.49
F127-PCL2000	1,460	600	1,856	1,696±31	8.82±1.65
F127-PCL3500	1,460	1,050	2,456	2,235±54	7.99±1.16
F127-PCL5300	1,460	1,590	2,516	2,321±29	7.92±1.16

Supporting information 4

1 Drawing the Linear Curve of Surfactant HLB Value

Table S4

The HLB and R-value of different kinds of surfactants ($\bar{x} \pm S$, n=3)

Name	H_w	H_o	R	HLB
Span-60	1.000	6.634±0.013	0.131±0.010	4.7
Span-20	1.000	1.961±0.021	0.338±0.018	8.6
EL-35	1.000	0.702±0.019	0.588±0.023	13.1
Tween-80	1.000	0.522±0.031	0.657±0.020	15.0
Tween-20	1.000	0.312±0.022	0.762±0.024	16.7
PEG-400	1.000	0.061±0.018	0.943±0.017	20.0

Note: H_w denotes relative coefficients of hydrophilic groups. H_o denotes the relative coefficients of hydrophobic groups. R presents the ratio of hydrophilic groups in the amphiphilic polymer, and HLB denotes hydrophile-lipophile balance.

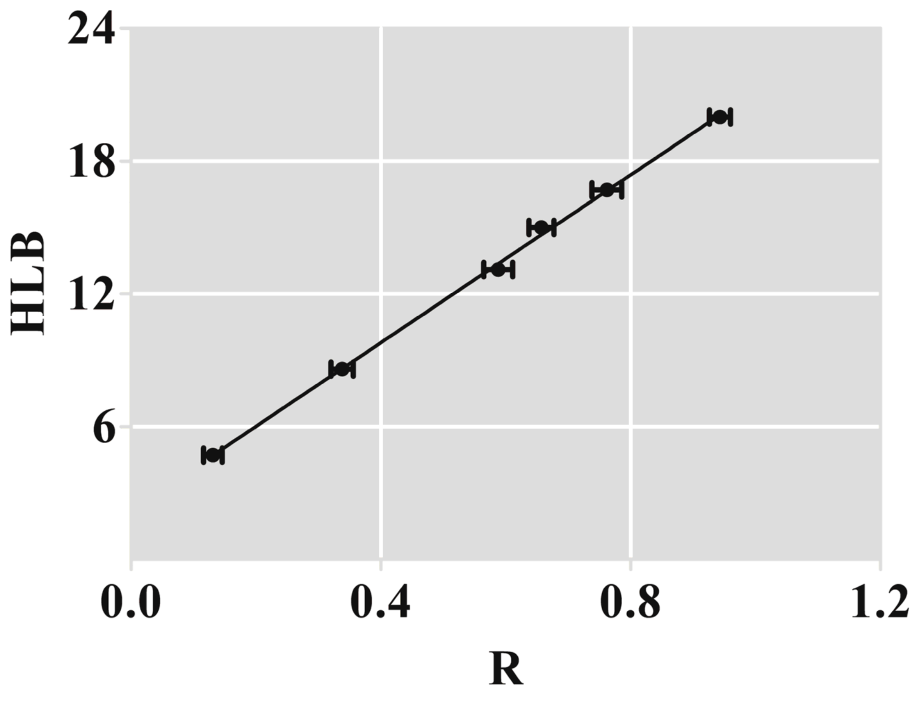

Figure S8. The linear relationship between HLB and R-value, R-value presents the ratio of hydrophilic groups in

the amphiphilic polymer, and HLB denotes hydrophile-lipophile balance.

2 Calculating the HLB of Raw Materials

Table S5

The HLB of raw materials ($\bar{x} \pm S$, n=3)

Group	MW	H_w	H_o	R	HLB
F127	14,600	1.000	0.168±0.002	0.856±0.001	18.434±0.025
	2,000	1.000	2.894±0.003	0.257±0.001	7.076±0.002
PCL	3,500	1.000	2.854±0.005	0.259±0.001	7.122±0.010
	5,300	1.000	2.828±0.002	0.261±0.001	7.154±0.006
	2,000	1.000	1.775±0.002	0.36±0.001	9.038±0.010
PLA	3,500	1.000	1.766±0.004	0.361±0.001	9.055±0.015
	5,000	1.000	1.755±0.002	0.363±0.001	9.086±0.001
PLGA	2,500	1.000	1.019±0.005	0.495±0.002	11.596±0.029
	4,700	1.000	1.022±0.003	0.495±0.001	11.580±0.011

Note: H_w denotes relative coefficients of hydrophilic groups, and H_o denotes relative coefficients of hydrophobic groups. R presents the ratio of the hydrophilic groups in the amphiphilic polymer, MW denotes molecular weight, and HLB denotes hydrophile-lipophile balance.

Supporting information 5

Table S6

The MCI and VMCI of different raw materials

Group	MCI					VMCI				
	$^0\chi$	$^1\chi$	$^2\chi$	$^3\chi$	$^4\chi$	$^0\chi^v$	$^1\chi^v$	$^2\chi^v$	$^3\chi^v$	$^4\chi^v$
F127	720.798	492.347	373.884	21.939	31.435	627.943	362.449	228.845	12.667	14.128
PCL-2000	98.388	64.445	48.059	3.255	4.427	87.823	54.956	36.366	1.066	1.462
PCL-3500	180.135	117.855	88.023	5.907	8.177	160.603	100.713	66.833	1.950	2.712
PCL-5300	256.431	167.705	125.322	8.382	11.677	228.532	143.419	95.269	2.775	3.879
PLA-2000	99.057	55.794	49.205	9.901	14.907	81.514	42.321	29.376	4.781	5.649
PLA-3500	172.380	97.403	86.237	17.219	28.147	142.285	74.031	52.812	8.266	9.903
PLA-5000	242.211	137.031	121.505	24.188	39.670	200.162	104.230	74.394	11.585	13.954
PLGA-2500	117.438	67.652	52.304	9.232	18.671	93.918	48.696	30.347	4.277	6.014
PLGA-4700	221.357	127.867	98.818	17.156	35.169	177.514	92.223	57.470	8.101	11.393

Note: $^m\chi_k$ denotes MCI; $^m\chi_k^V$ denotes VMCI; the order of the MCI is denoted by m; k denotes the type of the fragment of the molecule, for example path (p), cluster (c), and path-cluster (pc). The types of fragments used in this work are shown in Figure S3. The number of fragments of order *m* is denoted by n_m.

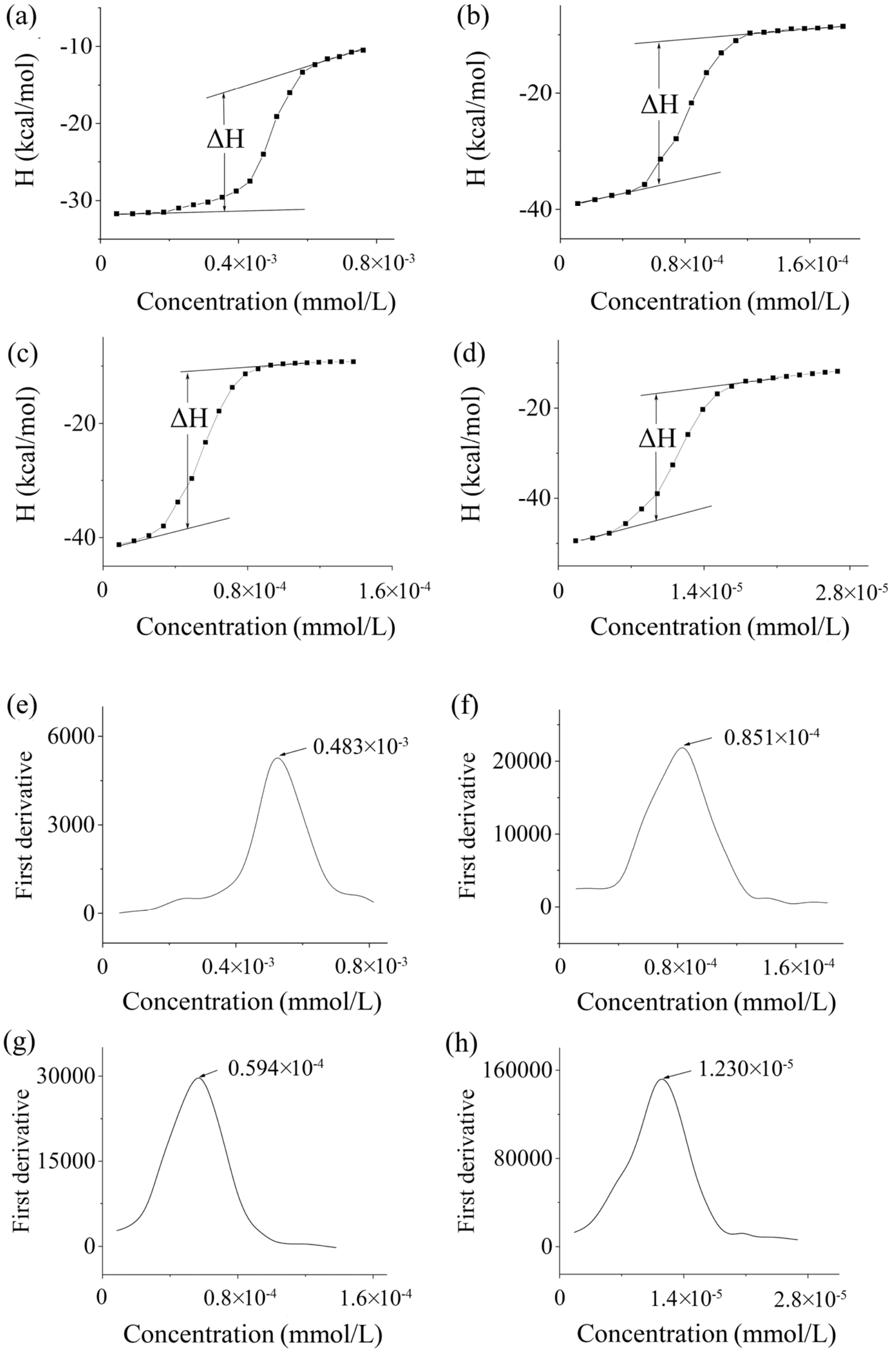

Figure S9. The calorimetry curves of F127-PLGA2500 (a), F127-PLGA4700 (b), F127-PLA5000(c), and F127-PCL5300 (d). ΔH denotes the enthalpy change in titration. The first derivative of the calorimetry curves of F127-PLGA2500 (e), F127-PLGA4700 (f), F127-PLA5000(g), and F127-PCL5300 (h). The arrows in the figure point out the CMC of these polymers.

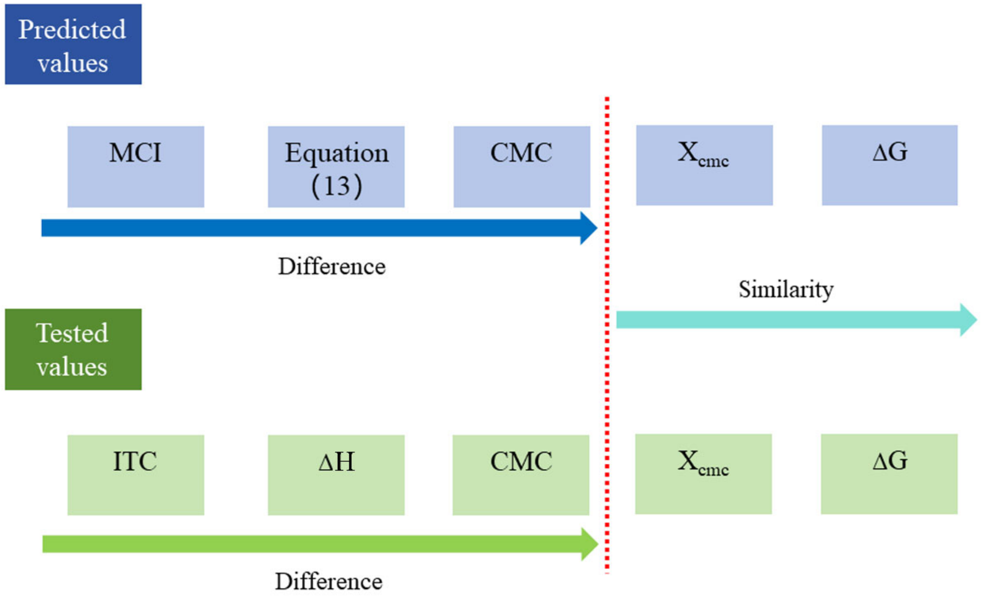

Figure S10. The approach obtains predicted and tested ΔG.

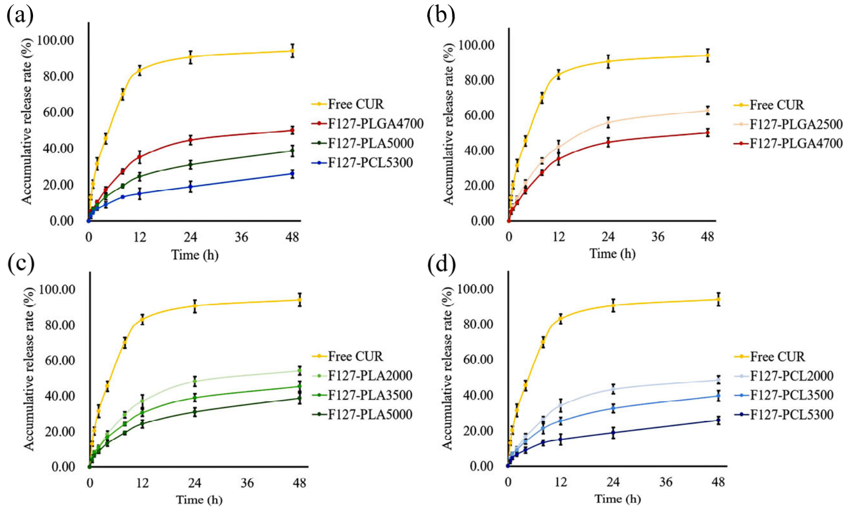

Figure S11. The accumulative release curves of free CUR and eight CUR loading micelles. (a) the release curves of CUR-loaded micelles prepared with PLGA, PLA, and PCL modified F127, which have similar molecular weight, (b) the release curve of CUR-loaded micelles prepared with PLGA modified F127, (c) the release curve of CUR-loaded micelles prepared with PLA modified F127, (d) the release curve of CUR-loaded micelles prepared with PCL modified F127.
